# Influence of Neuromuscular Training Interventions on Jump-Landing Biomechanics and Implications for ACL Injuries in Youth Females: A Systematic Review and Meta-analysis

**DOI:** 10.1007/s40279-025-02190-w

**Published:** 2025-04-17

**Authors:** Akhilesh Kumar Ramachandran, Jason S. Pedley, Sylvia Moeskops, Jon L. Oliver, Gregory D. Myer, Hung-I. Hsiao, Rhodri S. Lloyd

**Affiliations:** 1 Youth Physical Development Centre, Cardiff School of Sport and Health Sciences, Cardiff Metropolitan University, Cyncoed Campus, Cyncoed Road, Cardiff, CF23 6XD UK; 2 Sport Performance Research Institute, New Zealand (SPRINZ), AUT University, Auckland, New Zealand; 3 Centre for Sport Science and Human Performance, Waikato Institute of Technology, Hamilton, New Zealand; 4 Emory Sports Performance and Research Center (SPARC), Flowery Branch, GA USA; 5 Emory Sports Medicine Center, Atlanta, GA USA; 6 Department of Orthopaedics, Emory University School of Medicine, Atlanta, GA USA; 7 Wallace H. Coulter Department of Biomedical Engineering, Georgia Institute of Technology and Emory University, Atlanta, GA USA; 8 The Micheli Center for Sports Injury Prevention, Waltham, MA USA

## Abstract

**Background:**

Various exercise interventions are recommended to reduce the risk of anterior cruciate ligament (ACL) injury in females. However, the extent to which these training interventions influence lower-limb landing biomechanics in youth female remains unclear.

**Objective:**

This systematic review and meta-analysis aimed to quantitatively summarise the effectiveness of various training interventions on jump-landing biomechanics in youth females.

**Methods:**

We systematically searched PubMed, SPORTDiscus, EMBASE and Scopus. Articles were included if they: (1) conducted research on uninjured youth females (reported mean age < 18 years) with no restriction on playing level/experience or physical activity level; (2) performed any form of training intervention for ≥ 4 weeks; (3) reported any lower-limb kinematic (flexion/extension, adduction/abduction or internal/external rotation angles) or kinetic (joint moments or vertical ground reaction forces) data during the landing phase of jump-landing tasks, pre- and post-training intervention for both experimental and control groups, using a two- or three-dimensional motion capture system; (4) were randomised- or non-randomised controlled trials. The quality of the randomised controlled trials was assessed using the Risk of Bias tool 2, whereas the Downs and Black checklist was used for assessing the quality of non-randomised controlled trials. A multi-level meta-analytical model was used for conducting the quantitative analysis.

**Results:**

Thirteen studies (7 randomised controlled, 6 non-randomised controlled studies) involving 648 female participants were included in the final analyses. With regards to the overall quality of the included studies, three studies had high risk of bias while ten studies had some concerns. As part of the meta-analysis, we were able to analyse seven kinematic variables and two kinetic variables in aggregate. Compared with controls, the experimental group had significantly increased peak knee flexion angle (*g* = 0.58, *p* = 0.05) and reduced knee valgus motion (*g* =  − 0.86, *p* = 0.05) post-intervention. The effects on other kinematic and kinetic variables ranged from trivial to moderate and were not significantly altered as a result of various training interventions.

**Conclusion:**

The findings from the synthesised literature indicate that training interventions have small to moderate effects on peak knee flexion angle and knee valgus motion during jumping tasks. However, further research employing more consistent study designs and methodologies is required to better understand the changes in jump-landing biomechanics in the youth female population following training interventions.

**Supplementary Information:**

The online version contains supplementary material available at 10.1007/s40279-025-02190-w.

## Key Points

**Table:** 

Training interventions can increase peak knee flexion angle and reduce knee valgus motion during jump-landing tasks in youth females.
Since movements around hip and ankle can influence knee biomechanics during jump-landing tasks, it is recommended that future research should consider the influence of training interventions on these two joints as well.
There is a need for higher-quality research to better understand the influence of training interventions on lower-limb biomechanics during jump landing tasks in youth females.

## Introduction

An anterior cruciate ligament (ACL) rupture can be a debilitating and life-altering injury, resulting in a prolonged period of rehabilitation for athletes [1–3], difficulty returning to pre-injury performance levels [4, 5] and substantial treatment costs [6, 7]. There is evidence in the existing research literature that the incidence of ACL injuries and related surgical interventions such as ACL reconstruction has been increasing in the youth population [8, 9]. In particular, ACL injury is more prevalent in female than male athletes [10, 11] during non-contact scenarios such as single- and double-leg jump-landing tasks [12–14]. Differences in anatomy [15], joint biomechanics [16], hormones [17, 18], neuromuscular control [19, 20] and sociocultural and environmental factors such as limited access to facilities, training opportunities [21] and development staff and resources [21] are potential explanations for higher injury risk in females. In young athletes, the risk of secondary ACL injury is reported to be as high as 25–35% within 2–5 years of the first injury [22–24]. Further, some youth with ACL injury experience early onset of osteoarthritis within 15 years of the injury [25–27]. Therefore, establishing evidence-based strategies and targeted training interventions to reduce the risk and burden of ACL injury, especially in youth females, is of paramount importance in paediatric sports medicine.

Previous research has focussed on examining the association between landing biomechanics and risk of individuals sustaining an ACL injury [28–30]. For instance, studies have shown that female athletes landing from unilateral and bilateral jumps with lower peak knee flexion angle, higher peak knee abduction angle and moment, and higher vertical ground reaction force (vGRF) are at higher risk of ACL injury [31–34]. However, the findings in the current literature regarding the kinematic and kinetic variables that can be considered as risk factors for ACL injury are contradictory and inconclusive [28, 29, 35], warranting further research. Existing evidence indicates that training interventions can help in improving landing biomechanics by altering lower-limb flexion, adduction, rotation angles and moments, and landing GRF, which in turn could reduce ACL loading and injury risk [36–41]. For instance, 6 weeks of plyometric training for high-school female athletes (15 years old) can reduce their landing force from a vertical jump by 1.2 times their bodyweight [36]. Further, 8 weeks of basic resistance training for high-school female athletes (14.5 years old) can increase peak knee flexion angle (63° versus 70.9°) [42]. Various neuromuscular training programmes (hereon referred to as injury prevention programmes) combining plyometrics, strength training, and technique and balance training also have been developed to elicit adaptations of the neuromuscular system, which can improve landing biomechanics and reduce risk of ACL injury [27, 43–45]. These multimodal injury prevention programmes can reduce injury risk in female athletes by 45–67% [24, 46]. Despite the availability of various training programmes, ACL injury rates are increasing, particularly in younger children [8, 9, 47, 48]. A better understanding of the effects of various training programmes on jump-landing biomechanics could help refine and develop more effective training interventions.

Although a plethora of systematic reviews and meta-analyses have been conducted to determine the effectiveness of training interventions on ACL injury risk, most studies have examined the effects of interventions on injury incidence rate [49–52]. Further, previous reviews exploring the effects of training interventions on landing biomechanics have primarily been conducted in female athletes [41, 53–56], but with limited emphasis on how these interventions affect landing mechanics of youth females (< 18 years of age). Further research in this area can provide practitioners with novel information regarding the magnitude of changes in biomechanical variables induced by various training interventions during jump-landing tasks within this specific population, which in turn can influence training programme design. Therefore, the aim of the current meta-analysis is to quantitatively summarise the effectiveness of various training interventions on jump-landing biomechanics in youth females.

## Methods

This review was conducted in accordance with Preferred Reporting Items for Systematic Review and Meta-analyses (PRISMA) guidelines [57].

### Literature Search

A systematic literature search was conducted across the following scientific databases to identify original research articles published from inception to December 2023: PubMed, SPORTDiscus, EMBASE and Scopus. The Boolean operators ‘AND’ and ‘OR’ were used to combine various search terms. The complete search strategy is provided as Supplementary Appendix S1. The reference lists of included studies were screened by one author (A.K.R.) to identify any additional studies relevant for this review. Further, the reference lists of all relevant systematic reviews and meta-analyses that had reported biomechanical variables during jump landing tasks in youth population were checked by one author (A.K.R.) to identify any relevant studies for this review.

### Administration of the Systematic Review

One author (A.K.R.) carried out the search across all relevant databases. All articles were exported to Endnote (version 10), and any duplicate articles were removed. Articles were screened according to the inclusion/exclusion criteria. A three-stage process was followed to identify relevant articles. Articles were included in the first stage if they investigated biomechanics related to ACL injuries pre- and post-training intervention during jump-landing tasks. In the second stage, the abstract of each study was screened to exclude studies that did not report findings on female participants. The third stage involved reviewing the full text of all relevant studies that satisfied the eligibility criteria for their suitability for final inclusion. Two authors (A.K.R and R.S.L.) independently performed all these tasks. Potential discrepancies regarding inclusion/exclusion of studies were discussed and resolved between the two authors, consulting with an additional author (J.L.O.) as needed to reach consensus.

### Eligibility Criteria

Inclusion criteria were based on the population, intervention, comparator, outcome and study design (PICOS) concept, whereby studies needed to have: (1) conducted research on uninjured youth females (reported mean age < 18 years) with no restriction on playing level/experience or physical activity level; (2) performed a training intervention such as resistance, plyometric or injury prevention programmes for ≥ 4 weeks; (3) reported any lower-limb kinematic (flexion/extension, adduction/abduction or internal/external rotation angles) or kinetic (joint moments or vertical ground reaction forces) data during the landing phase of jump-landing tasks, pre- and post-training intervention for both the experimental group and control group, using a two- or three-dimensional motion capture system; (4) been randomised controlled trials or non-randomised controlled trials (hereon referred to as ‘controlled trials’) that provided pre- and post-training intervention values for kinematic or kinetic variables for both experimental and control groups.

Exclusion criteria were as follows: (1) studies that did not include females or did not report biomechanical outcomes separately for females in the event of mixed samples (males and females); (2) studies that particularly recruited female participants with a biomechanical risk factor associated with ACL injury (e.g. increased knee abduction angle) prior to starting the intervention; (3) studies that reported biomechanical variables during non-jump-landing tasks, such as side-stepping or cutting; (4) studies in which no biomechanical variables were reported during jump-landing tasks; (5) book chapters, narrative or scoping reviews, systematic reviews and meta-analyses, conference proceedings, poster presentations, pilot studies, conference abstracts, reviews, clinical commentaries, theses and dissertations; and (6) articles not published in English.

### Risk of Bias Assessment

The methodological quality of randomised controlled trials (RCTs) included in our review was assessed using the Cochrane Collaboration Risk of Bias Tool version 2 [58]. Included studies were assessed according to the following domains: (i) randomisation process; (ii) deviations from intended interventions; (iii) missing outcome data; (iv) measurement of the outcome; and (v) selection of the reported result. Each domain was judged individually as low, some concerns, or high risk. Studies were classified as "low risk” if low risk of bias was demonstrated for all domains, and “high risk” if high risk of bias was demonstrated in at least one domain [58]. Studies were classified as “some concerns” if there were some concerns regarding bias in one or more than one domain but not substantial enough to be classified as high risk of bias [58]. Figures for the assessment were created using ‘robvis’ [59], an online tool for summarising the findings from the risk of bias assessment.

For controlled trials (CTs) in which the experimental and control groups were not randomised, the Downs and Black checklist was used to assess risk of bias [60]. The checklist consists of 27 items that address methodological components, including external validity, internal validity (bias and confounding variables) and power. The quality index of the checklist has high criterion validity (*r* = 0.90), internal consistency (KR-20 = 0.89), and test–retest (*r* = 0.88) and inter-rater (*r* = 0.75) reliability [60]. Power-related items were modified from a scale of 0–5 to a binary scale, with all items given a score of 0 (no) or 1 (yes). Points were converted to a percentage score, with studies classified as low risk of bias (≥ 71%), some concerns (51–70%) or high risk of bias (≤ 50%). Two authors (A.K.R. and R.S.L.) independently assessed the quality of the included studies, consulting with a third author (J.L.O.) in cases of disagreement to reach consensus.

### Certainty of Evidence

We used the five Grading of Recommendations Assessment, Development, and Evaluation (GRADE) criteria (i.e. risk of bias, inconsistency, indirectness, imprecision, and publication bias) to assess certainty of evidence for each outcome [61]. Since our analysis included studies in which experimental and control groups were or were not randomised, the overall level of evidence was downgraded by one level and initially rated as moderate. Each domain was then further downgraded for the following limitations: total sample size of < 800 (imprecision); overall *I*^2^ > 40% (inconsistency); risk of bias if > 50% of studies reporting a particular variable were rated as having high risk of bias or some concerns; or publication bias if influential analysis revealed any outliers for a particular variable. Certainty of evidence for all kinematic and kinetic variables is presented in Supplementary Table S1.

### Data Extraction and Reduction

The following data were extracted from included articles: (1) author name and year of publication; (2) age, stature and body mass of participants; (3) sporting activity and level; (4) jump-landing tests used in the study; (5) mode of data collection; (6) analysed kinematic and kinetic data; (7) units of measurement for reported variables; and (8) mean and standard deviation for each biomechanical variable across both experimental and control groups.

Sign conventions were inconsistent among included studies and thus were standardised as follows [62]: positive values for hip flexion, knee flexion, ankle dorsiflexion, hip adduction, knee adduction, hip internal rotation angles and moments. Phases of interest included discrete values at initial foot contact and/or peak values. Joint range of motion was defined as the difference in joint angles at the frame prior to initial contact and maximum flexion. Joint moments were reported as external moments, and studies that reported the unit of moments as Nm or Nm/kg were included in the meta-analysis.

### Handling Missing and Duplicate Data

In cases of missing information in the included studies, the first or corresponding authors were contacted via e-mail to provide information regarding samples size, pre- and post-training intervention means, and standard deviation (SD) scores. If the authors did not respond to the first e-mail, a follow-up email was sent after 1 week. Studies were excluded from analysis if no response was obtained after two attempts or if the authors were unable to provide the required data. In instances where data were reported in graphical format, WebPlotDigitiser (https://automeris.io/WebPlotDigitizer/) was used to derive the numerical value, as this has been demonstrated to be a valid procedure (*r* = 0.99, *p* < 0.001) [63]. In cases where standard error (SE) was reported, SD was obtained using the following formula: SD = SE × sqrt(*N*), where *N* is the sample size of the respective group (experimental or control) [64]. Two studies by the same author reported identical data for the same variable and group in different publications [65, 66]. In this case, duplicate data were removed, and the analysis was conducted using the single record.

### Meta-analysis

Meta-analysis for biomechanical variables was conducted using R software with the metafor [67] and clubSandwich [68] packages. Each kinematic and kinetic variable recorded at initial contact or peak value during the landing phase of the jumping task was considered for meta-analysis if it was reported in a minimum of three studies irrespective of the study quality. Included studies reported repeated measures for outcomes during various jump-landing tasks for the same participants. To account for the likelihood of statistical dependency for this hierarchical structure, the overall effect size for kinematic and kinetic variables was calculated using a multi-level, mixed effects meta-analysis with robust variance estimation [69]. Such an approach allows exploration of the heterogeneity present across multiple levels (within- and between-group variance) and provides a robust method for meta-analysis while taking into consideration the dependency of effect estimates derived from common samples [70]. To account for correlation between effect estimates, variance was replaced with the entire “V matrix” [69, 71], indicating the variance–covariance matrix of estimates. Block-diagonal covariance matrices were estimated with an assumed correlation of *r* = 0.5 [68]. No included studies provided correlation coefficient (*r*) values between pre- and post-intervention measures. Therefore, a sensitivity analysis using different values (*r* = 0.5; *r* = 0.7; and *r* = 0.8) was performed. As the results of the meta-analyses did not vary when different *r* values were used, we used a conservative estimate of *r* = 0.7 in our analysis [72, 73]. The restricted maximum-likelihood estimation method was used to estimate model parameters. The overall effect size (Hedges’ *g*) for kinematic and kinetic findings was calculated. Effect sizes were set as trivial (< 0.20), small (0.21–0.60), moderate (0.61–1.20), large (1.21–2.00), very large (2.01–4.00) and extremely large (> 4.00) [74].

Uncertainty in meta-analysis estimates was expressed using 95% confidence intervals (95% CIs), representing ranges of values compatible with our models and assumptions. In addition, we also calculated 95% prediction intervals (95% PIs) to convey the likely range of true measurement properties in similar future studies [75]. To describe the extent of heterogeneity, we calculated *Q*-statistics as well as restricted maximum-likelihood estimates of within- (tau^2^) and between-studies (tau^3^) variances (SD; tau) and the *I*^2^ of within- and between-studies variances. *I*^2^ implies the percentage of variance due to study heterogeneity rather than sampling error, with values of 0–25% indicating trivial, 25–50% indicating low, 50–75% indicating moderate and 75–100% indicating high heterogeneity [74].

### Influential Analysis

To assess the influence of each effect size on the summary effect and heterogeneity, Cook’s distance analysis [76] and Baujat plots [77] were obtained. We used Cook’s distance values greater than three times the mean [78], and potential outliers were excluded from the dataset to examine whether there were substantial changes in summary estimates. Effect sizes (with and without outliers) were reported for variables in case outliers were detected. Funnel plots were visually inspected for asymmetry and publication bias.

## Results

### Search Results and Selection

**Fig. 1:**
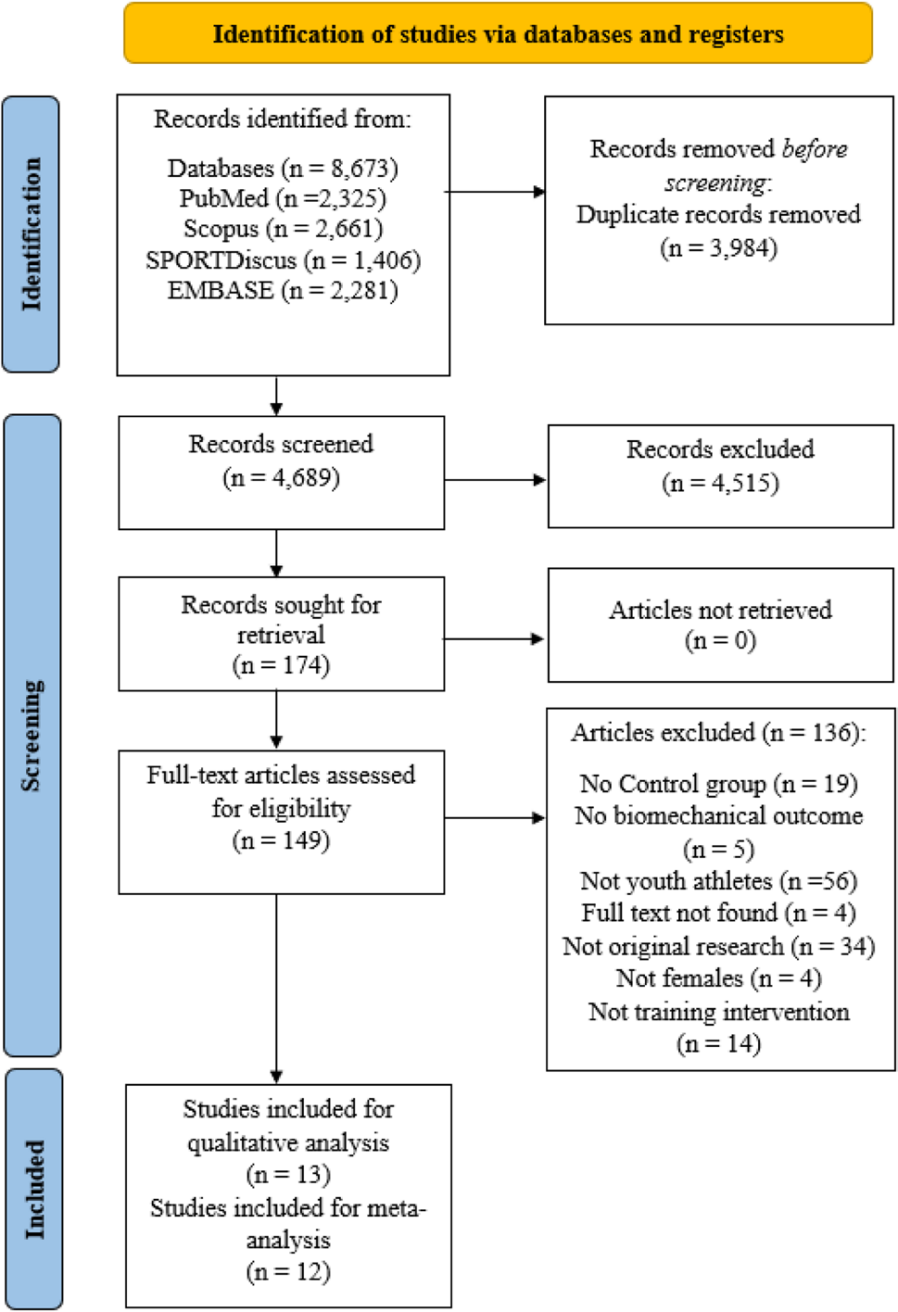
PRISMA flowchart of the study selection process

The electronic database and manual search yielded 8673 articles, from which 3984 articles were removed as duplicate studies and 4537 articles were excluded based on title and abstract screening. A total of 149 full-text articles were further screened, from which 16 studies met full inclusion criteria. However, data could not be obtained for three studies, which were thus were excluded. Overall, 13 studies were included for qualitative analysis and 12 studies were included for meta-analysis (Fig. 1) [5, 65, 66, 79–88].

### Study Characteristics

Our review encompassed a total of 648 participants (351 in experimental group, 297 in control group). The mean age, stature and weight of participants were 14.48 ± 1.59 years, 1.62 ± 0.08 m and 54.31 ± 8.75 kg, respectively. Seven of the 13 studies reported the maturity status of the athletes. Of the 480 participants included in these studies, 135 were classified as pre-pubertal, 141 as mid/late-pubertal and 110 as post-pubertal. Further, one study classified 51 participants as pre-adolescent (10–12 years) [65], and another study classified 43 participants as adolescent (14–18 years) [66]. Participants competed in sporting activities such as basketball [79, 83–85], soccer [5, 65, 66, 79, 80], volleyball [79, 86], field hockey [79], handball [87], netball [81] and dancing [88]. Only one study [82] recruited participants who were not involved in any sporting activity. The sporting level of participants varied from recreational [88], high school [83–85], and club level [5, 65, 66, 81] to elite [87], but this information was not provided in three studies [79, 80, 86]. Of the 13 studies included in our review, 6 studies were RCTs [5, 79, 81–83, 86], 1 study was a cluster RCT [80] and 6 studies were CTs [65, 66, 84, 85, 87, 88]. A detailed description of participant characteristics is presented in Table 1.

**Table 1:** Participant characteristics from the included studies

Study	Study design	Sample size	Age (years)	Height (m)	Weight (kg)	Sporting activity	Sporting level	Kinematic data collection method	Kinetic data collection method
Brown et al. [79]	RCT	EG1: 10 EG2: 7 EG3: 13 CG:13	EG1: 14.1 ± 1.2 EG2: 15 ± 0.6 EG3: 14.8 ± 0.6 CG: 14.7 ± 2.6	EG1: 1.63 ± 0.08 EG2: 1.66 ± 0.07 EG3: 1.64 ± 0.02 CG: 1.62 ± 0.06	EG1: 50.6 ± 8.5 EG2: 61.7 ± 11.2 EG3: 59.5 ± 8.9 CG: 53.9 ± 10.1	Basketball, field hockey, soccer, volleyball	NR	3D motion capture system: 8 cameras (Vicon; 240 fps)	Dual force plates (AMTI Corp; 1200 Hz)
De Ste Croix et al. [80]	Cluster RCT	EG: 71 CG: 54	EG: 13.1 ± 1.7 CG: 12.8 ± 1.6	EG: 1.56 ± 0.09 CG: 1.54 ± 0.09	EG: 49.5 ± 10 CG: 51.4 ± 9.6	Soccer	NR	2D: High-speed video cameras (Quintic)	–
Hopper et al. [81]	RCT	EG: 13 CG: 10	12.2 ± 0.9	EG: 1.64 ± 0.07 CG: 1.64 ± 0.1	EG: 50.7 ± 8.8 CG: 53.3 ± 8.2	Netball	Local club	3D: 10 cameras (Vicon; 250 Hz)	Single 600 × 900 mm force platform (Kistler; 1000 Hz)
Katsikari et al. [82]	RCT	EG: NR CG: NR	9–11	EG: 1.45 ± 0.0 8 CG: 1.43 ± 0.06	EG: 38.5 ± 8.1 CG: 38.5 ± 3.7	Untrained	–	3D: 6 cameras (Vicon; 100 Hz)	Single 40 × 60 cm force plate (Bertec Corp; 1000 Hz)
Lim et al. [83]	RCT	EG: 11 CG: 11	EG: 16.2 ± 1.2 CG: 16.1 ± 1	EG: 1.72 ± 0.05 CG: 1.71 ± 0.08	EG: 64.2 ± 6.1 CG: 64 ± 7.3	Basketball	High school	2D: 6 video cameras (Panasonic)	Dual force plates (AMTI Corp; 1200 Hz)
Otsuki et al. [84]	CT	EG: EP: 18 LP: 28 PP: 33 CG: EP: 17 LP: 22 PP: 36	EG: EP: 12.9 ± 0.7 LP: 13.8 ± 1 PP: 15.9 ± 0.7 CG: EP: 12.6 ± 0.6 LP: 14 ± 0.9 PP: 16 ± 0.6	EG: EP: 1.51 ± 0.05 LP: 1.61 ± 0.06 PP: 1.61 ± 0.07 CG: EP: 1.51 ± 0.05 LP: 1.61 ± 0.05 PP: 1.62 ± 0.04	EG: EP: 41.9 ± 4.6 LP: 52.6 ± 6.7 PP: 54.4 ± 6.9 CG: EP: 40.1 ± 4.6 LP: 51.7 ± 5.7 PP: 55.6 ± 5.4	Basketball	High school	2D: 3 video cameras (Casio Exilim; 30 Hz)	–
Otsuki et al. [85]	CT	EG: 32 CG: 28	EG: 13.1 ± 0.8 CG: 13.1 ± 0.8	EG: 1.56 ± 0.07 CG: 1.57 ± 0.08	EG: 47 ± 7 CG: 46.7 ± 8.7	Basketball	High school	2D: 3 video cameras (Casio Exilim; 30 Hz)	–
Rojano Ortega et al. [86]	RCT	EG: 14 CG: 14	EG: 16.1 ± 1.1 CG: 15.7 ± 0.7	EG: 1.67 ± 0.02 CG: 1.66 ± 0.05	EG: 67.8 ± 5.5 CG: 61.3 ± 7.2	Volleyball	NR	-	Single force plate (Kistler; 500 Hz)
Schmidt et al. [87]	CT	EG: 12 CG: 7	EG: 15.8 ± 0.4 CG: 16.0 ± 1.3	EG: 1.73 ± 0.05 CG: 1.74 ± 0.07	EG: 71.5 ± 10.2 CG: 68.5 ± 10.4	Handball	Elite	3D: 12 cameras (Qualisys; 120 Hz)	Dual force plates (AMTI Corp; 1000 Hz)
Sudds et al. [88]	CT	EG: 9 CG: 11	14–17	NR	NR	Dancing	Recreational	3D: 8 cameras (Motion Analysis Corp; 240 Hz)	Dual force plates (Kistler; 2400 Hz)
Taghizadeh Kerman et al. [5]	RCT	EG: 18 CG: 18	EG: 11.1 ± 1.2 CG: 11.2 ± 1.2	EG: 1.49 ± 0.05 CG: 1.48 ± 0.06	EG: 41.4 ± 3.5 CG: 40.5 ± 3.5	Soccer	Local club	3D: 8 cameras (Qualisys; 200 Hz)	–
Thompson et al. [65]	CT	EG: 28 CG: 23	EG: 11.8 ± 0.8 CG: 11.2 ± 0.6	EG: 1.54 ± 0.08 CG: 1.49 ± 0.08	EG: 41.6 ± 8.5 CG: 38.1 ± 6	Soccer	Local club	3D: 8 cameras (Motion Analysis Corp; 200 Hz)	Three force plates (Bertec Corp; 2000 Hz)
Thompson-Kolesar et al. [66]	CT	EG: Pre-: 28 Post-: 22 CG: Pre-: 23 Post-: 21	EG: Pre-: 11.8 ± 0.8 Post-: 15.9 ± 0.9 CG: Pre-: 11.2 ± 0.6 Post-: 15.7 ± 1.1	EG: Pre-: 1.54 ± 0.08 Post-: 1.66 ± 0.04 CG: Pre-: 1.49 ± 0.08 Post-: 1.66 ± 0.06	EG: Pre-: 41.6 ± 8.5 Post-: 58.2 ± 5.6 CG: Pre-: 38.1 ± 6 Post-: 57.7 ± 7.7	Soccer	Local club	3D: 8 cameras (Motion Analysis Corp; 200 Hz)	Three force plates (Bertec Corp; 2000 Hz)

Abbreviations: CG, control group; CT, controlled trial; EG, experimental group; EP, early pubertal; LP, late pubertal; NR, not reported; PP, post pubertal; RCT, randomised controlled trial

Articles included in our review aimed to examine jump-landing biomechanics of youth females pre- and post-training interventions. Seven studies had participants perform the bilateral drop vertical jump [65, 66, 81, 82, 84, 85, 87], four studies used the single-leg drop jump [5, 65, 66, 87], two studies used single-leg [80, 88] and double-leg countermovement jumps/vertical jump test [83, 86, 88], one study used a broad jump with single-leg landing test [81], and one study [79] used a unilateral jump test (i.e. participants jumped forward landing only on their left foot and jumped laterally to the right, or jumped forward landing only on their right foot and jumped laterally to the left). The same study [79] also used a bilateral test in which participants jumped forward with both feet and performed a maximum vertical jump. For the single-leg drop jump test, one study [87] had participants perform only the landing without a subsequent jump. With regards to the drop jump test, the height from which participants were asked to jump was predominantly 30 cm [5, 65, 66, 87] and 31 cm [81, 84, 85], although one study used 20-, 35- and 50-cm boxes [82]. Kinematic data were collected using three-dimensional motion capture systems in nine studies [5, 65, 66, 79, 81–83, 87, 88], while three studies [80, 84, 85] used two-dimensional video cameras. One study did not report any kinematic data [86].

With regards to the training interventions, 11 studies implemented an injury prevention programme [5, 65, 66, 79–81, 83–85, 87, 88], 3 studies used plyometric training [79, 82, 86], and 1 study [79] implemented core and balance training. Of the ten studies that performed injury prevention programmes, one study implemented the FIFA 11 + injury prevention programme [5], one study implemented the FIFA 11 + injury prevention programme for dancers [88] while two studies implanted the revised FIFA Medical and Research Centre (F-MARC) or FIFA 11 + injury prevention programme [65, 66]. The various training components implemented in the remaining seven studies [79–81, 83–85, 87] were dynamic warm-up [80, 81, 83], strength training [79, 81, 83–85, 87], plyometric training [79–81, 83–85, 87], balance training [79, 87], speed training [79], change of direction (COD)/agility training [80, 83–85], core training [79], jump and landing exercises [87] and dynamic flexibility [80, 83]. Duration of the training interventions was 6–24 weeks, with a frequency of 1–3 sessions/week. All details related to training interventions and outcomes reported in the included studies are presented in Table 2. A detailed description of individual exercises performed in the included studies is presented in Supplementary Table S2.

**Table 2:** Training intervention characteristics and outcome measures reported in included studies

Study	Type of training intervention	Frequency (sessions/week)	Duration of intervention (weeks)	Sets × (reps, time, or distance)	Jumping test performed	Kinematic outcomes reported	Kinetic outcomes reported
Brown et al. [79]	EG1: Injury prevention programme EG2: Core + balance training EG3: Plyometric training	3	6	(1–3) × [(4–30 reps) or (6–30 s)]	**Unilateral jump:** Jump forward landing only on left foot and jump laterally to the right, or vice versa **Bilateral jump:** Jump forward on both feet and perform maximum vertical jump	**Hip:** Peak flexion and adduction angles **Knee:** Peak flexion and abduction angles	**Hip:** Peak flexion and adduction moments **Knee:** Peak flexion and abduction moments
De Ste Croix et al. [80]	Injury prevention programme	3 (1 supervised; 2 unsupervised)	16	NR × [(15–20 m) or (1–25 reps) or (10–60 s)]	**Unilateral:** Single-leg countermovement jump	**Knee:** Flexion range of motion (initial contact–peak); abduction motion	**Knee:** Peak abduction moment
Hopper et al. [81]	Injury prevention programme	3	6	3 × (5–8 reps)	**Bilateral:** Drop vertical jump (31 cm) **Unilateral:** Broad jump with single-leg landing	**Hip:** Flexion at initial contact and peak flexion angles; abduction at initial contact and peak abduction angles; external rotation at initial contact and peak external rotation angles **Knee:** Flexion at initial contact and peak flexion angles; abduction at initial contact and peak abduction angles; external rotation at initial contact and peak external rotation angles	Peak vertical ground reaction force
Katsikari et al. [82]	Plyometric training	2	10	60–140-foot contacts	**Bilateral:** Drop vertical jump (20, 35, and 50 cm)	**Knee:** Peak knee flexion angle	**Knee:** Peak flexion moment
Lim et al. [83]	Injury prevention programme		8	(2–3 or 30 s) × (1–20 reps) or (50–100 yd)	**Bilateral:** Vertical jump	**Knee:** Peak flexion angle; internal rotation at initial contact and peak internal rotation angles	**Knee:** Peak knee extension and abduction moments
Otsuki et al. [84]	Injury prevention programme	3	24	(1–2) × (10–20 reps)	**Bilateral:** Drop vertical jump (31 cm)	**Knee:** Flexion range of motion (initial contact–peak); abduction motion	**Knee:** Peak abduction moment
Otsuki et al. [85]	Injury prevention programme	3	24	(1–2) × (10–20 reps)	**Bilateral:** Drop vertical jump (31 cm)	**Knee:** Flexion range of motion; abduction motion/medial knee displacement	**Knee:** Peak abduction moment
Rojano Ortega et al. [86]	Plyometric training	2	7	(2–3) × (8–15 reps)	**Bilateral:** Countermovement jump	NR	Peak vertical ground reaction force
Schmidt et al. [87]	Injury prevention programme	1	7	NR	**Bilateral:** Drop vertical jump (30 cm) **Unilateral:** Single-leg drop vertical jump (30 cm)	**Knee:** Flexion at initial contact and peak flexion angles; abduction at initial contact and peak abduction angles; internal rotation at initial contact and peak internal rotation angles	**Knee:** Peak flexion, abduction and internal rotation moments Peak vertical ground reaction force
Sudds et al. [88]	FIFA 11 + injury prevention programme for dancers	3	8	(1–2) × ((2–12 reps) or (10–15 s))	**Bilateral:** Countermovement jump **Unilateral:** Single-leg countermovement jump	**Hip:** Peak flexion and adduction angles **Knee:** Peak flexion and abduction angles	**Hip:** Peak extension and abduction moments **Knee:** Peak extension and adduction moments
Taghizadeh Kerman et al. [5]	FIFA 11 + injury prevention programme	2	8	NR	**Unilateral:** Single-leg drop vertical jump (30 cm)	**Trunk:** Peak flexion, lateral flexion and rotation angles **Hip:** Peak adduction, flexion and internal rotation angles **Knee:** Peak flexion, abduction (peak) and rotation angles	NR
Thompson et al. [65]	F-MARC/FIFA 11 + injury prevention warm-up programme	2	7–8	NR	**Bilateral:** Drop vertical jump (30 cm) **Unilateral:** Single-leg drop vertical jump (30 cm)	**Hip:** Peak adduction angle **Knee:** Peak flexion and abduction angles **Ankle:** Peak eversion angle	**Hip:** Peak adduction moment **Knee:** Peak flexion and abduction moments **Ankle:** Peak eversion moment
Thompson-Kolesar et al. [66]	F-MARC/FIFA 11 + injury prevention warm-up programme	2	7–8	NR	**Bilateral:** Drop vertical jump (30 cm) **Unilateral:** Single-leg drop vertical jump (30 cm)	**Knee:** Abduction at initial contact and peak abduction angles	**Knee:** Abduction at initial contact and peak abduction moments

Abbreviations: EG, experimental group; F-MARC, FIFA Medical and Research Centre; GRF, ground reaction force; IC, initial contact; NR, not reported; ROM, range of motion; SL, single leg

### Methodological Quality Assessment

**Fig. 2:**
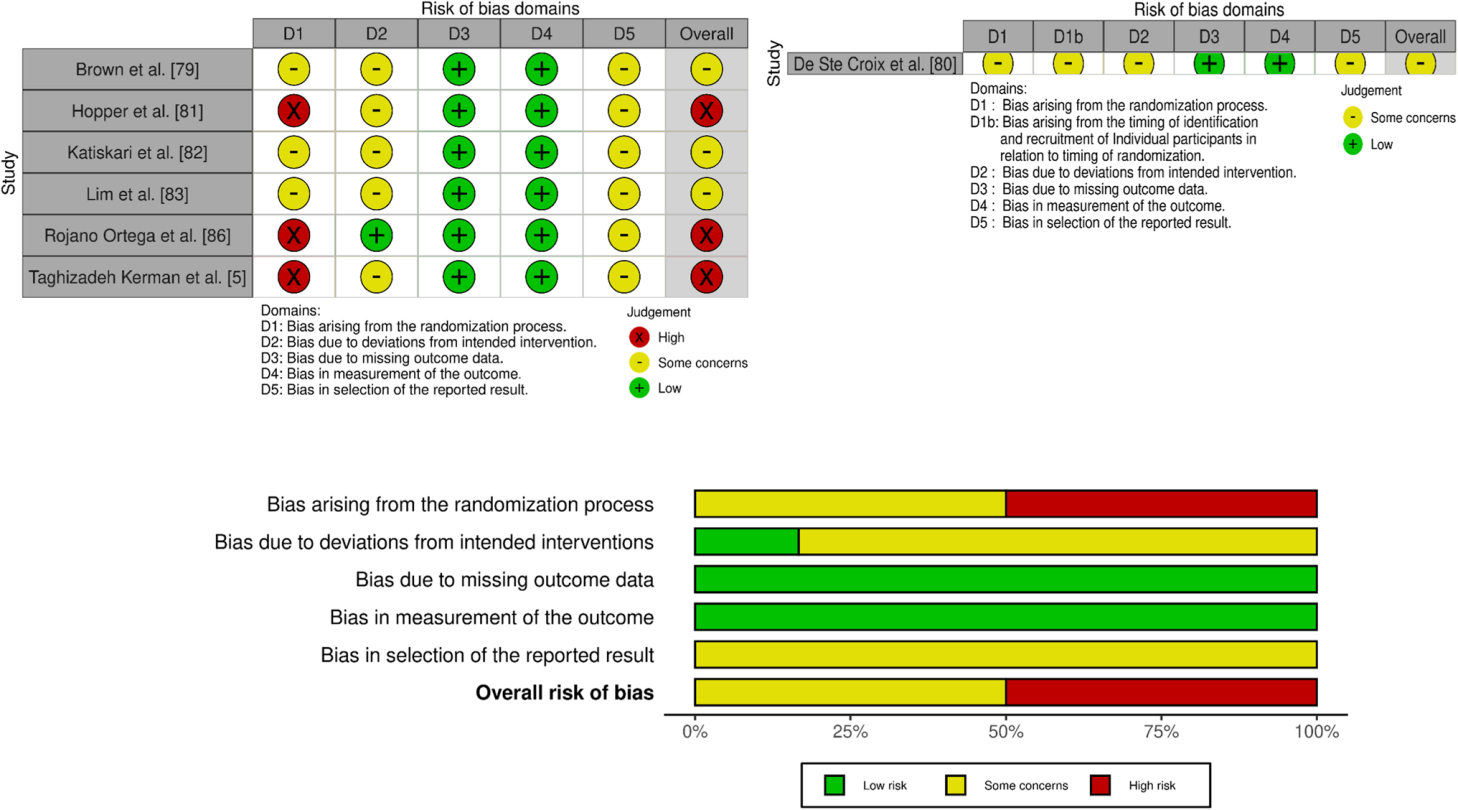
Risk of bias assessment results for randomised controlled studies (left) and cluster randomised controlled studies (right)

Of the six RCTs, three studies were classified as high of risk bias [5, 81, 86], and three studies had some concerns [79, 82, 83]. Additionally, one cluster RCT study had some concerns [80]. Individual domain ratings for each study are shown in Fig. 2.

With regards to the Downs and Black checklist, all six studies in which participants in experimental and control groups were not randomised were of moderate quality and had some concerns of bias [65, 66, 84, 85, 87, 88]. The overall methodological quality (mean ± SD) was 66 ± 4%, with a range of 64–71%. Detailed scoring for each study is presented in Table 3.

**Table 3:** Quality rating^a^ of controlled trials, per Downs and Black checklist [60]

Quality assessment	Reporting										External validity		
Study	1	2	3	4	5	6	7	8	9	10	11	12	13
Otsuki et al. [84]	1	1	1	1	1	1	1	0	1	1	0	1	1
Otsuki et al. [85]	1	1	1	1	1	1	1	0	1	1	0	1	1
Schmidt et al. [87]	1	1	1	1	1	1	1	0	1	1	0	1	1
Sudds et al. [88]	1	1	1	1	1	1	1	1	1	1	1	1	1
Thompson et al. [65]	1	1	1	1	0	1	1	0	1	1	0	1	1
Thompson-Kolesar et al. [66]	1	1	1	1	0	1	1	0	1	1	0	1	1

Quality assessment	Internal validity							Internal validity- confounding (selection bias)						Power	Results		
Study	14	15	16	17	18	19	20	21	22	23	24	25	26	27	Total score	Overall rating (in %)	Quality
Otsuki et al. [84]	0	0	1	1	1	1	1	0	1	0	0	0	1	1	19	68	Moderate
Otsuki et al. [85]	0	0	1	1	1	1	1	0	1	0	0	1	1	0	19	68	Moderate
Schmidt et al. [87]	0	0	1	1	1	1	1	0	1	0	0	0	1	0	18	64	Moderate
Sudds et al. [88]	0	0	1	1	1	1	1	0	1	0	0	0	1	0	20	71	Moderate
Thompson et al. [65]	0	0	1	1	1	1	1	0	1	0	0	0	1	0	17	61	Moderate
Thompson-Kolesar et al. [66]	0	0	1	1	1	1	1	0	1	0	0	0	1	0	17	61	Moderate

^a^Study rated as 2 points if resistance training, sport training experience, and maturity details provided; 1 point if any one provided; 0 if no information provided

### Meta-analysis Findings

#### Kinematic Findings

Meta-analysis was conducted on seven kinematic variables. Pooled estimates showed that there was a significant increase in peak knee flexion angle (*g* = 0.58) and decrease in knee valgus motion (*g* =  − 0.86) in the experimental group compared with controls. For the remaining variables, effect sizes ranged from trivial to small, and differences between experimental and control groups were non-significant. The certainty of evidence was very low for all kinematic variables.

##### Peak Hip Adduction Angle

**Fig. 3:**
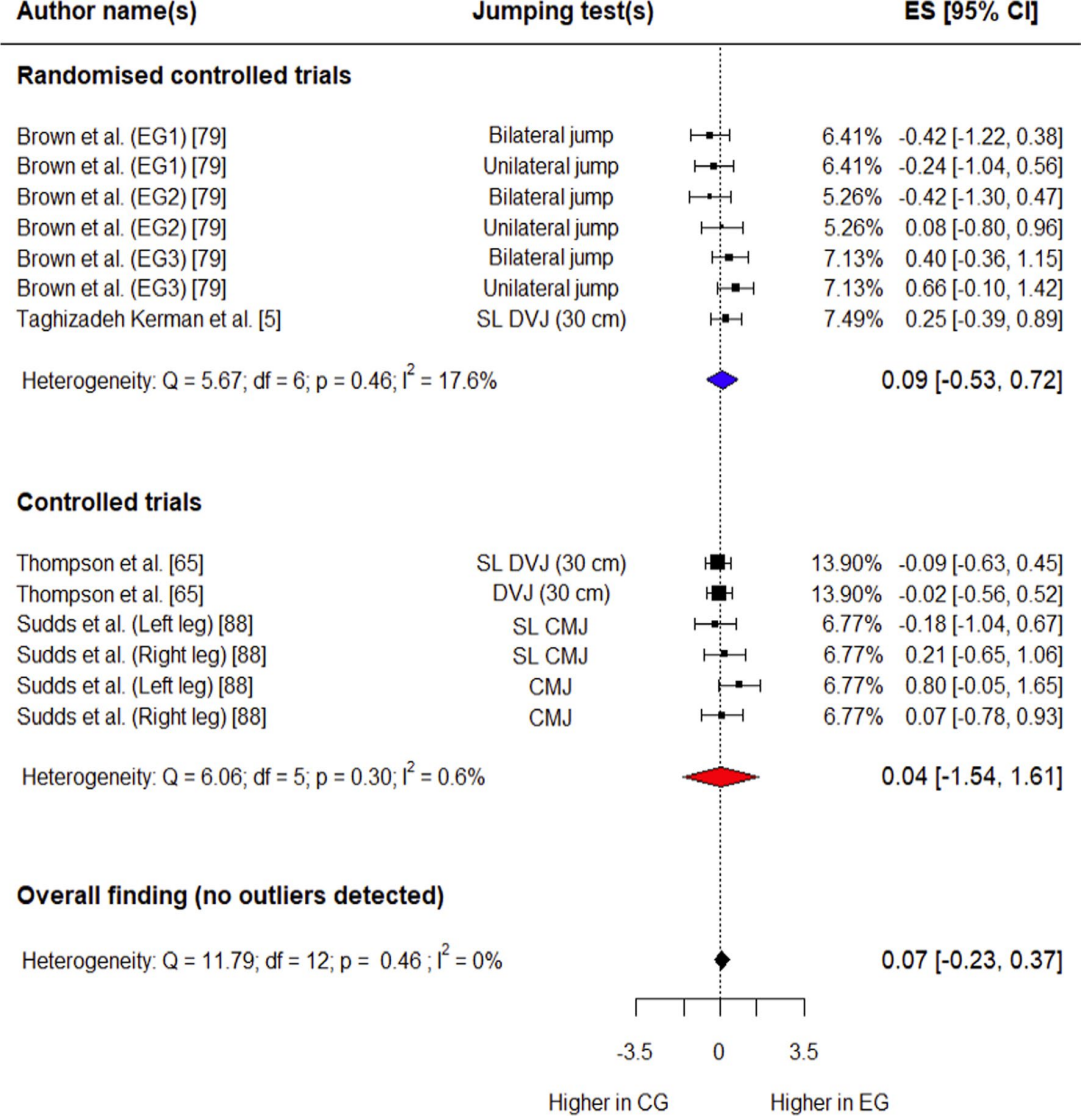
Forest plot showing effects of the training intervention on peak hip adduction angle (95% CI, 95% confidence interval; CG, control group; CMJ, countermovement jump; DVJ, drop vertical jump; EG, experimental group; ES, effect size; SL, single-leg)

Four studies (two RCTs [5, 79] and two CTs [65, 88]) involving 150 participants (85 in experimental group, 65 in control group) provided data for this outcome. The control group had a trivial, non-significant decrease in peak hip adduction angle compared with the experimental group (*g* = 0.07; 95% CI: − 0.23 to 0.37; 95% PI: − 0.23 to 0.37; *p* = 0.56) (Fig. 3). Overall heterogeneity was trivial (*I*^2^ = 0%), and no outliers were detected for this variable.

##### Peak Hip Flexion Angle

**Fig. 4:**
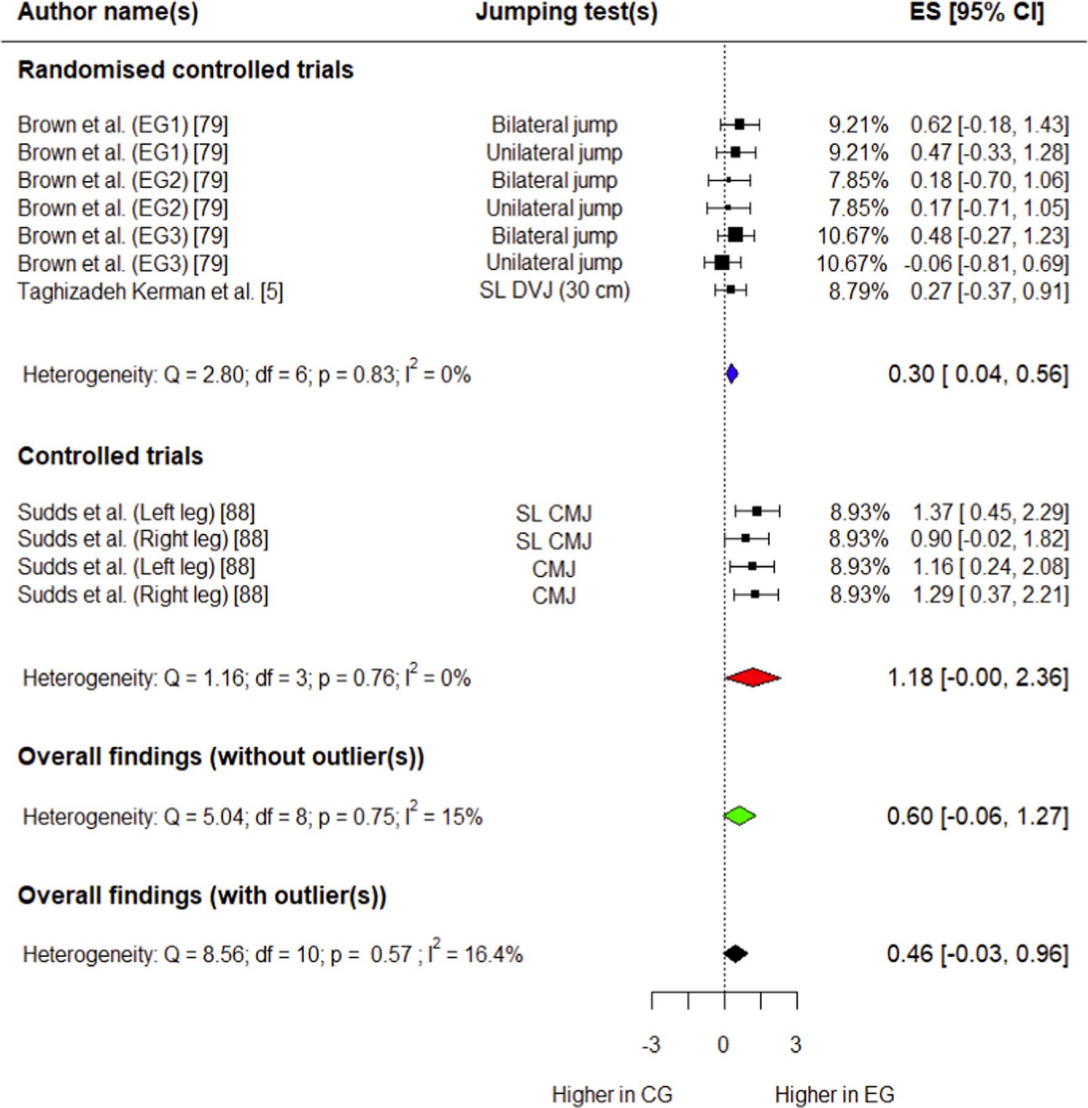
Forest plot showing effects of the training intervention on peak hip flexion angle (95% CI, 95% confidence interval; CG, control group; CMJ, countermovement jump; DVJ, drop vertical jump; EG, experimental group; ES, effect size; SL, single-leg)

Three studies (two RCTs [5, 79] and one CT [88]) involving 99 participants (57 in experimental group, 42 in control group) provided data for this outcome. The experimental group had a small, non-significant increase in peak hip flexion angle compared with controls (*g* = 0.46; 95% CI: − 0.03 to 0.96; 95% PI: − 0.25 to 1.18; *p* = 0.06). Overall heterogeneity was trivial (*I*^2^ = 16.4%) (Fig. 4). Outliers were detected during influential analysis (Supplementary Fig. S10), and effect size was calculated after removing these data points. The experimental group had a moderate, non-significant increase in peak hip flexion angle compared with controls (*g* = 0.60; 95% CI: − 0.06 to 1.27; 95% PI: − 0.29 to 1.49; *p* = 0.06) (Fig. 4). Overall heterogeneity was trivial (*I*^2^ = 15%).

##### Peak Knee Flexion Angle

**Fig. 5:**
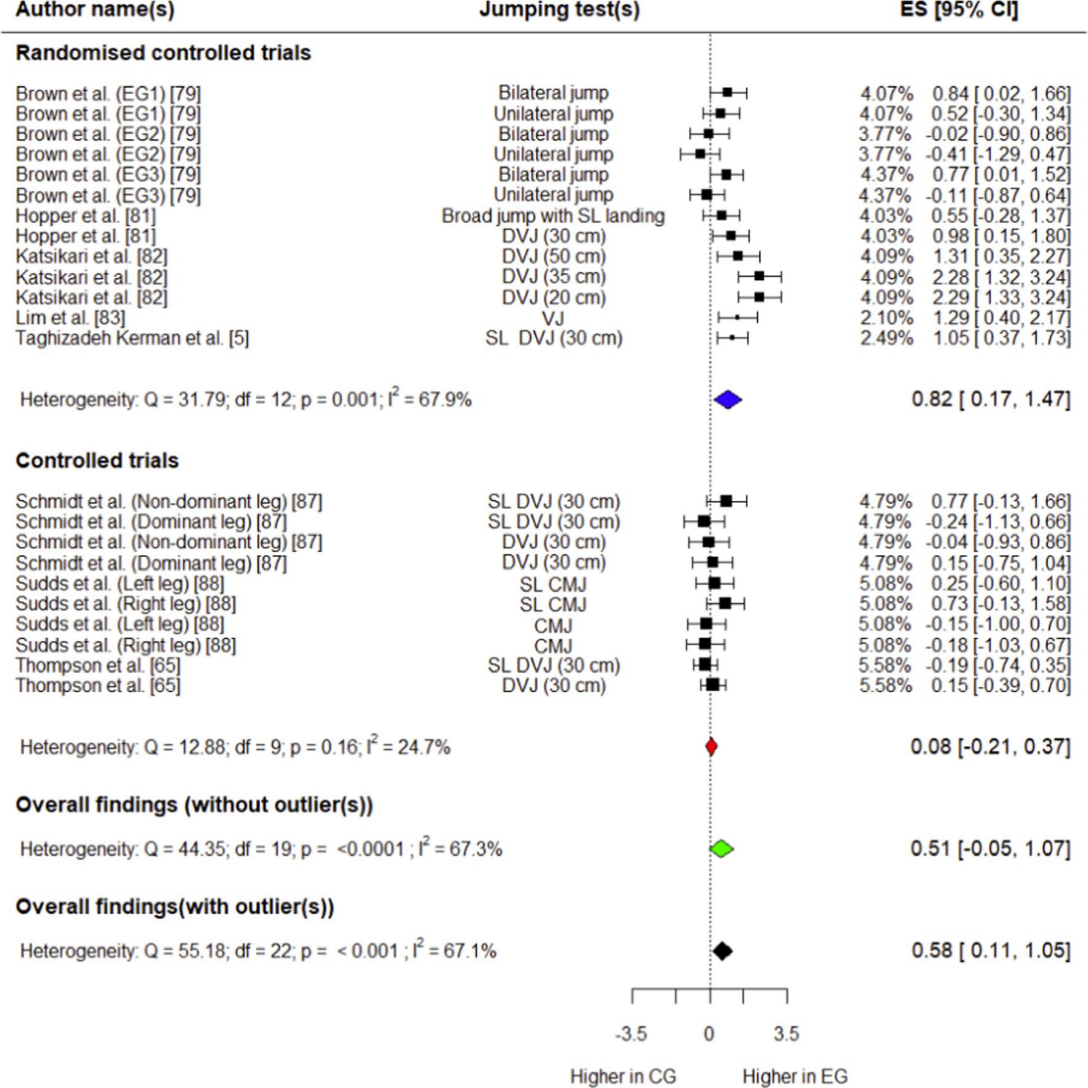
Forest plot showing effects of the training intervention on peak knee flexion angle (95% CI, 95% confidence interval; CG, control group; CMJ, countermovement jump; DVJ, drop vertical jump; EG, experimental group; ES, effect size; SL, single-leg; SL, single-leg; VJ, vertical jump)

Eight studies (five RCTs [5, 79, 81–83] and three CTs [65, 87, 88]) involving 238 (133 in experimental group, 105 in control group) participants provided data for this outcome. The experimental group had a small but significant increase in peak knee flexion angle compared with controls (*g* = 0.58; 95% CI: 0.11 to 1.05; 95% PI: − 0.83 to 1.98; *p* = 0.02) (Fig. 5). Overall heterogeneity was moderate for this variable (*I*^2^ = 67.1%). Outliers were detected during influential analysis (Supplementary Fig. S11), and effect size was calculated after removing these data points. The experimental group had a small, non-significant increase in peak knee flexion angle compared with controls (*p* > 0.05) (Fig. 5), and overall heterogeneity was moderate (*I*^2^ = 67.3%).

##### Peak Knee Abduction Angle

**Fig. 6:**
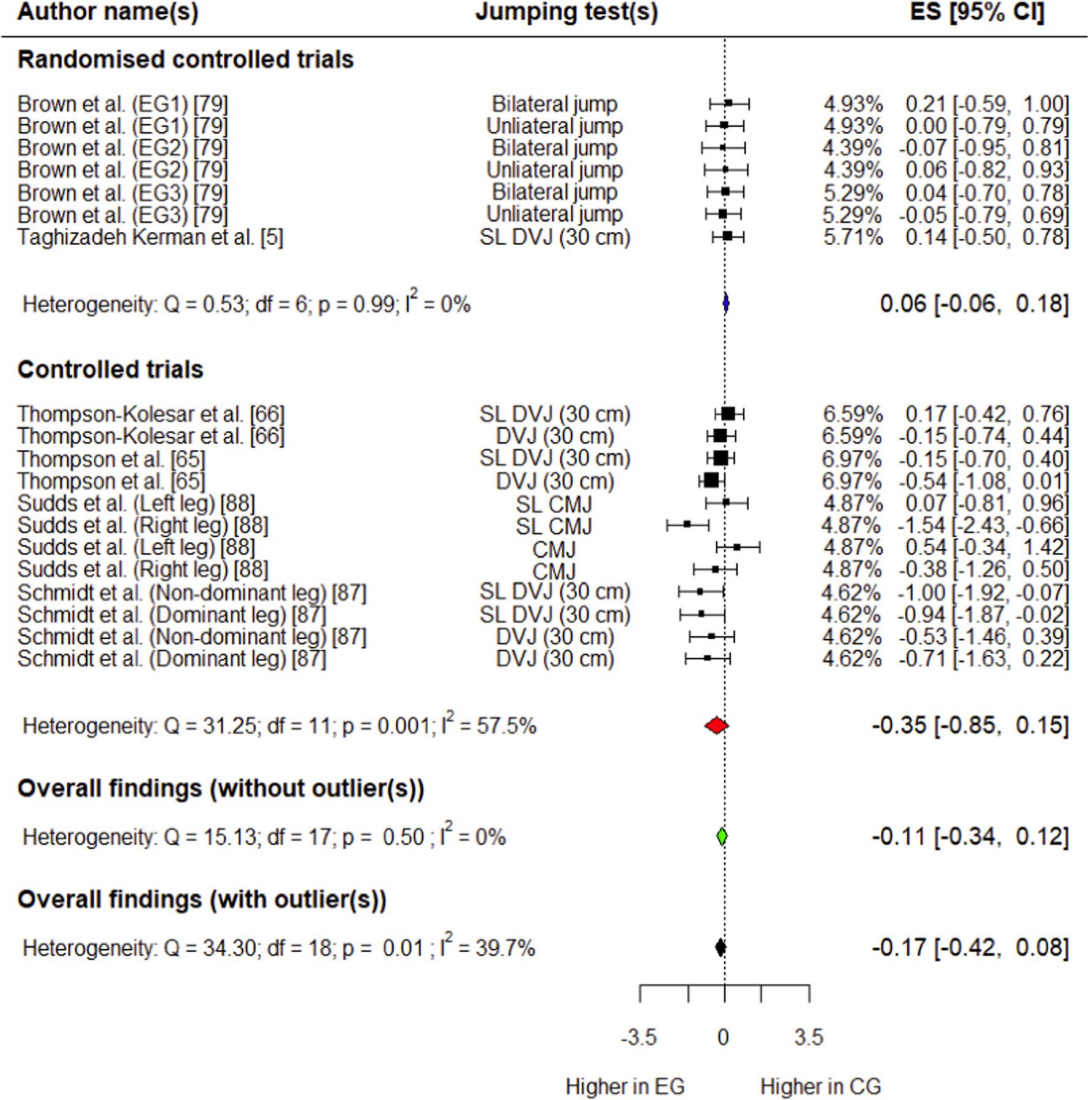
Forest plot showing effects of the training intervention on peak knee abduction angle (95% CI, 95% confidence interval; CG, control group; CMJ, countermovement jump; DVJ, drop vertical jump; EG, experimental group; ES, effect size; SL, single-leg)

Six studies (two RCTs [5, 79] and four CTs [65, 66, 87, 88]) involving 176 (101 in experimental group, 75 in control group) participants provided data for this outcome. The control group had a trivial, non-significant decrease in knee abduction angle compared with the experimental group (*g* =  − 0.17; 95% CI: − 0.42 to 0.08; 95% PI: − 0.95 to 0.61; *p* = 0.15) (Fig. 6). Overall heterogeneity was low (*I*^2^ = 39.7%). Outliers were detected during influential analysis (Supplementary Fig. S12), and the effect size was calculated after removing these data points. The control group had a trivial, non-significant decrease in peak knee abduction angle compared with the experimental group (*p* = 0.05) (Fig. 6), and overall heterogeneity was trivial (*I*^2^ = 0%).

##### Knee Abduction Angle at Initial Contact

**Fig. 7:**
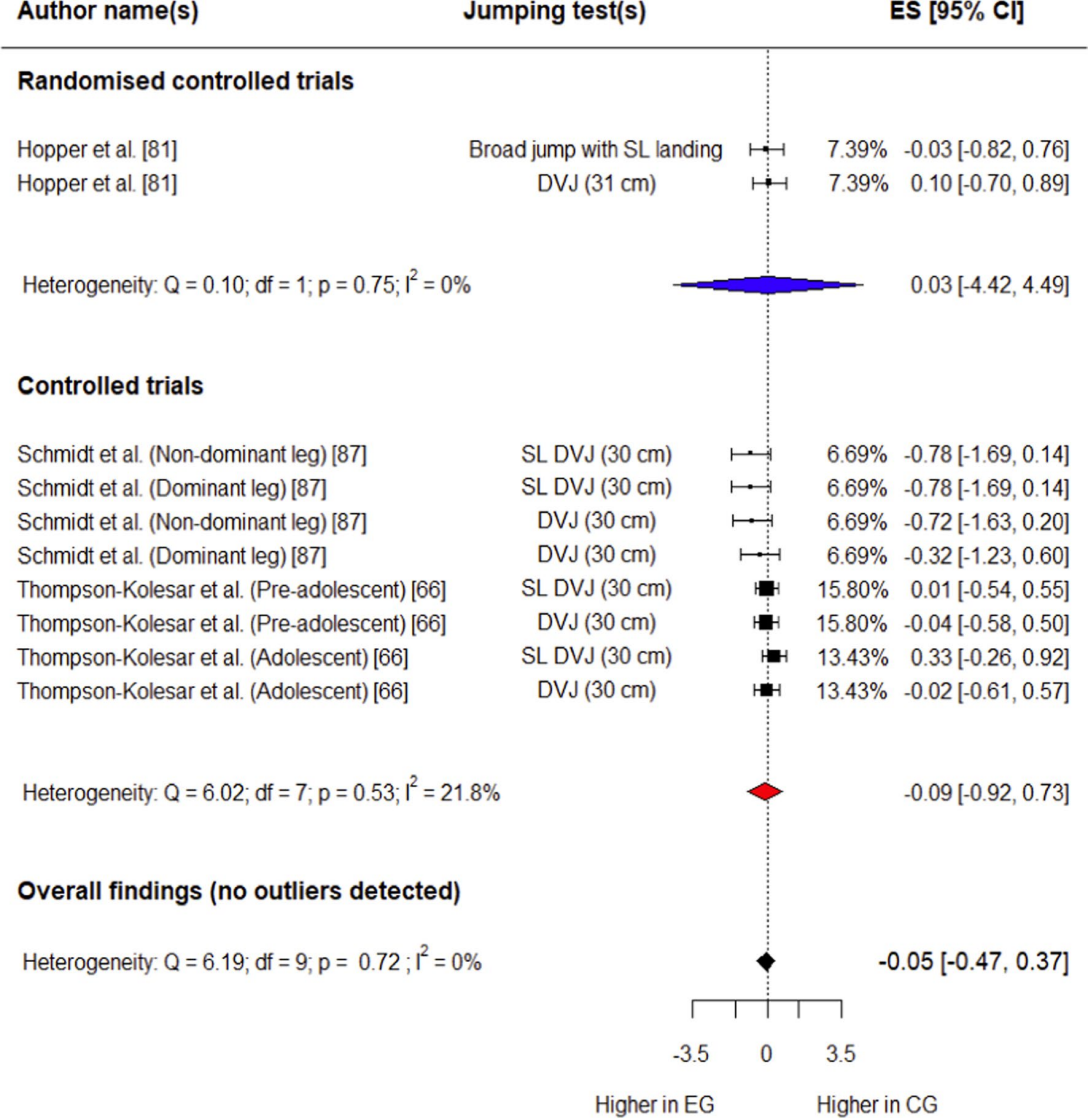
Forest plot showing effects of the training intervention on knee abduction angle at initial contact (95% CI, 95% confidence interval; CG, control group; DVJ, drop vertical jump; EG, experimental group; ES, effect size; SL, single-leg)

Three studies (one RCT [81] and two CTs [66, 87]) involving 136 participants (75 in experimental group, 61 in control group) provided data for this outcome. The control group had a trivial, non-significant decrease in knee abduction angle at initial contact compared with the experimental group (*g* =  − 0.05; 95% CI − 0.47 to 0.37; 95% PI − 0.47 to 0.37; *p* = 0.71) (Fig. 7). Overall heterogeneity was trivial (*I*^2^ = 0%). No outliers were detected for this variable.

##### Knee Flexion Range of Motion

**Fig. 8:**
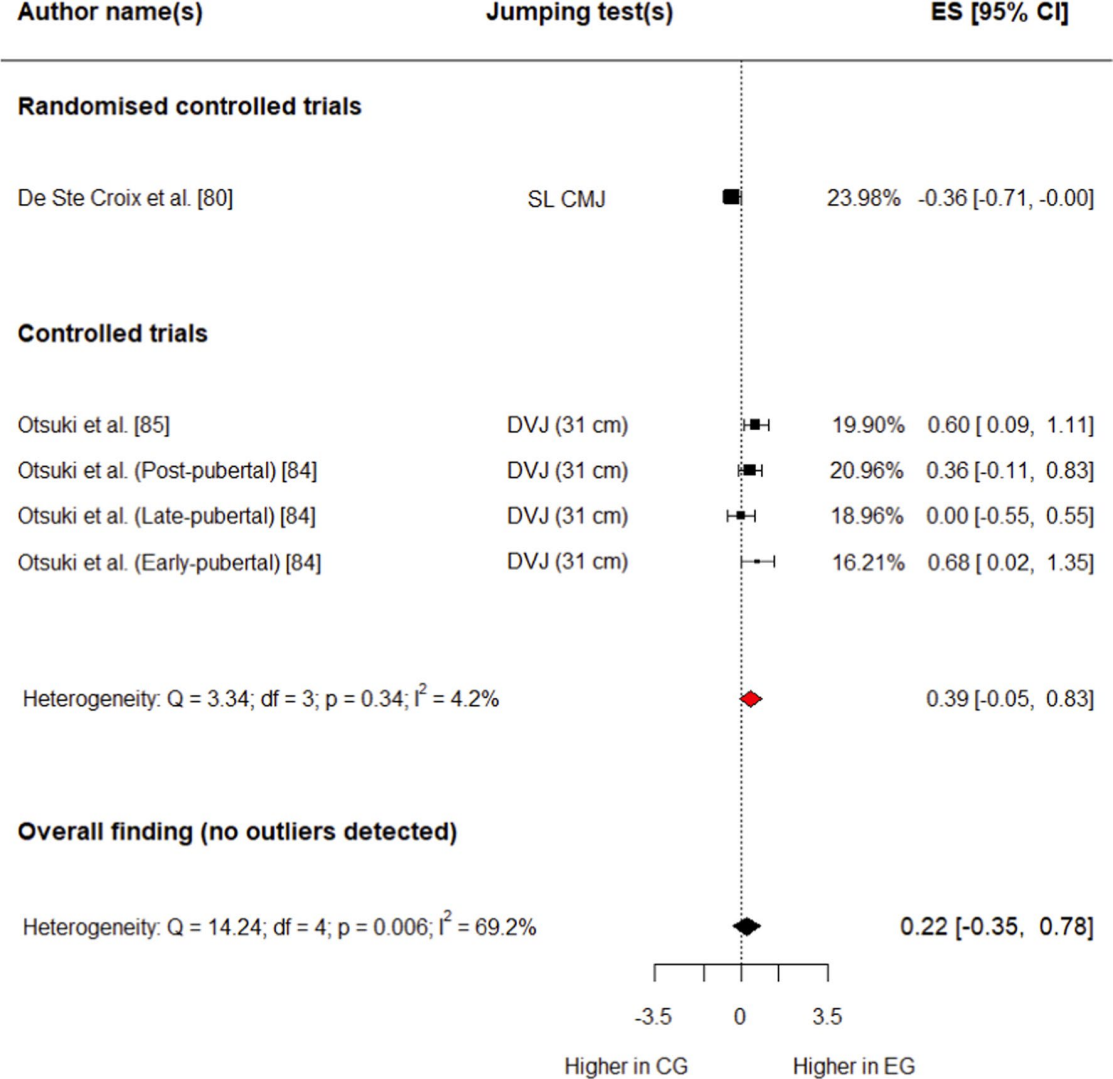
Forest plot showing effects of the training intervention on knee flexion range of motion (95% CI, 95% confidence interval; CG, control group; CMJ, countermovement jump; DVJ, drop vertical jump; EG, experimental group; ES, effect size; SL CMJ, single-leg)

Three studies (one RCT [80] and two CTs [84, 85]) involving 339 (182 in experimental group, 157 in control group) participants provided data for this outcome. The experimental group had a small, non-significant increase in knee flexion range of motion compared with the control group (*g* = 0.22; 95% CI − 0.35 to 0.78; 95% PI − 0.96 to 1.40; *p* = 0.34) (Fig. 8). Overall heterogeneity was moderate (*I*^2^ = 69.2%). No outliers were detected for this variable.

##### Knee Valgus Motion

**Fig. 9:**
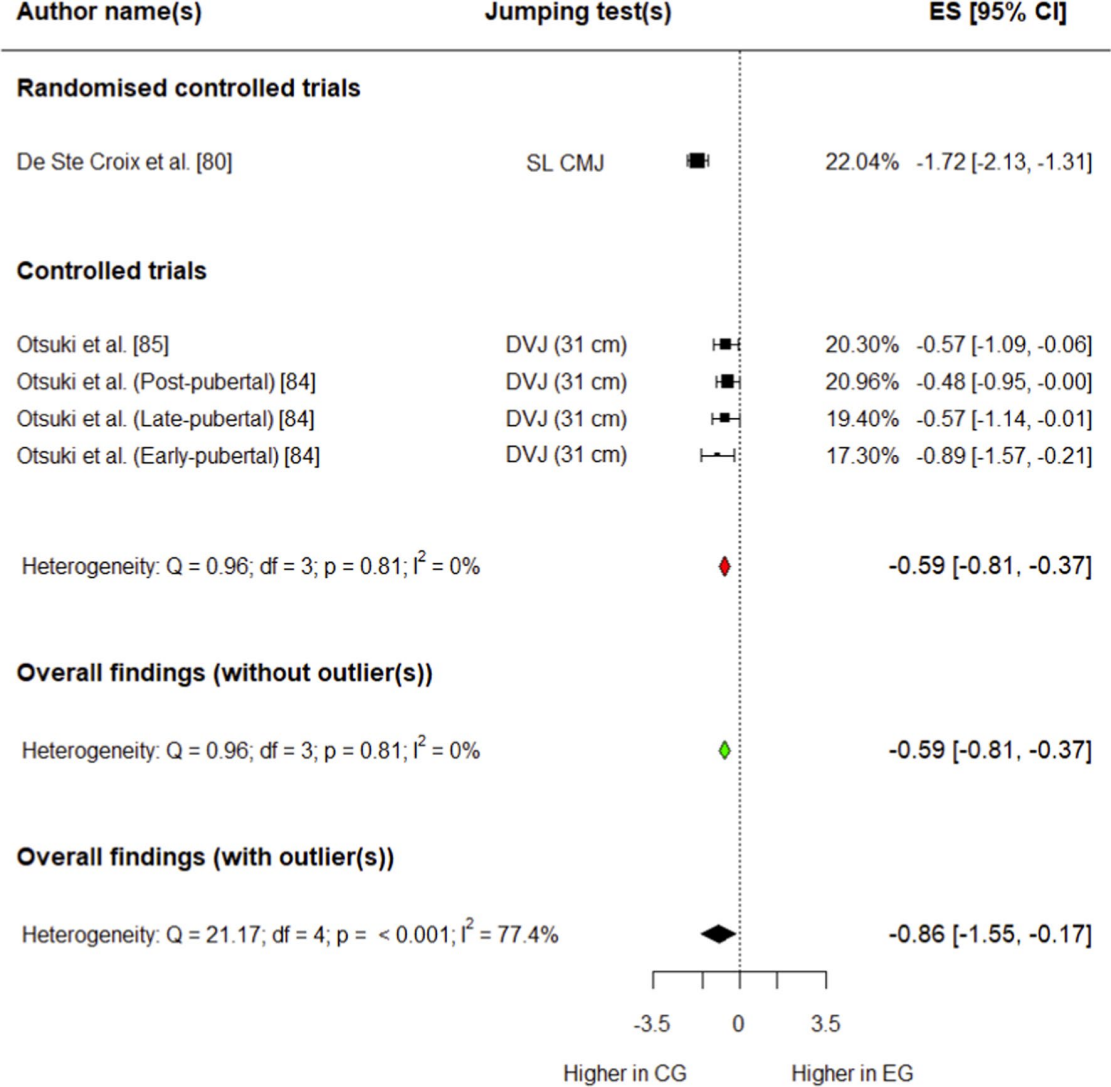
Forest plot showing effects of the training intervention on knee valgus motion (95% CI, 95% confidence interval; CG, control group; CMJ, countermovement jump; DVJ, drop vertical jump; EG, experimental group; ES, effect size; SL CMJ, single-leg)

Three studies (one RCT [80] and two CTs [84, 85]) involving 339 (182 in experimental group, 157 in control group) participants provided data for this outcome. The experimental group had a moderate, significant decrease in knee valgus motion compared with the control group (*g* =  − 0.86; 95% CI − 1.55 to − 0.17; 95% PI − 2.37 to 0.65; *p* = 0.03) (Fig. 9). Overall heterogeneity was high for this variable (*I*^2^ = 77.4%). Outliers were detected during influential analysis (Supplementary Fig. S13), and the effect size was calculated after removing these data points. The experimental group had a small, significant decrease in knee valgus motion compared with the control group (*g* =  − 0.59; 95% CI/PI − 0.81 to − 0.37; *p* = 0.004) (Fig. 9). Overall heterogeneity was trivial after influential analysis (*I*^2^ = 0%).

#### Kinetic Findings

Our meta-analysis examined two kinetic variables. Overall effect estimates were found to be trivial for both variables. Kinetic variables had outliers, and analyses were conducted after removing these data points. Certainty of evidence was very low for all kinetic variables.

##### Peak Knee Flexion Moment

**Fig. 10:**
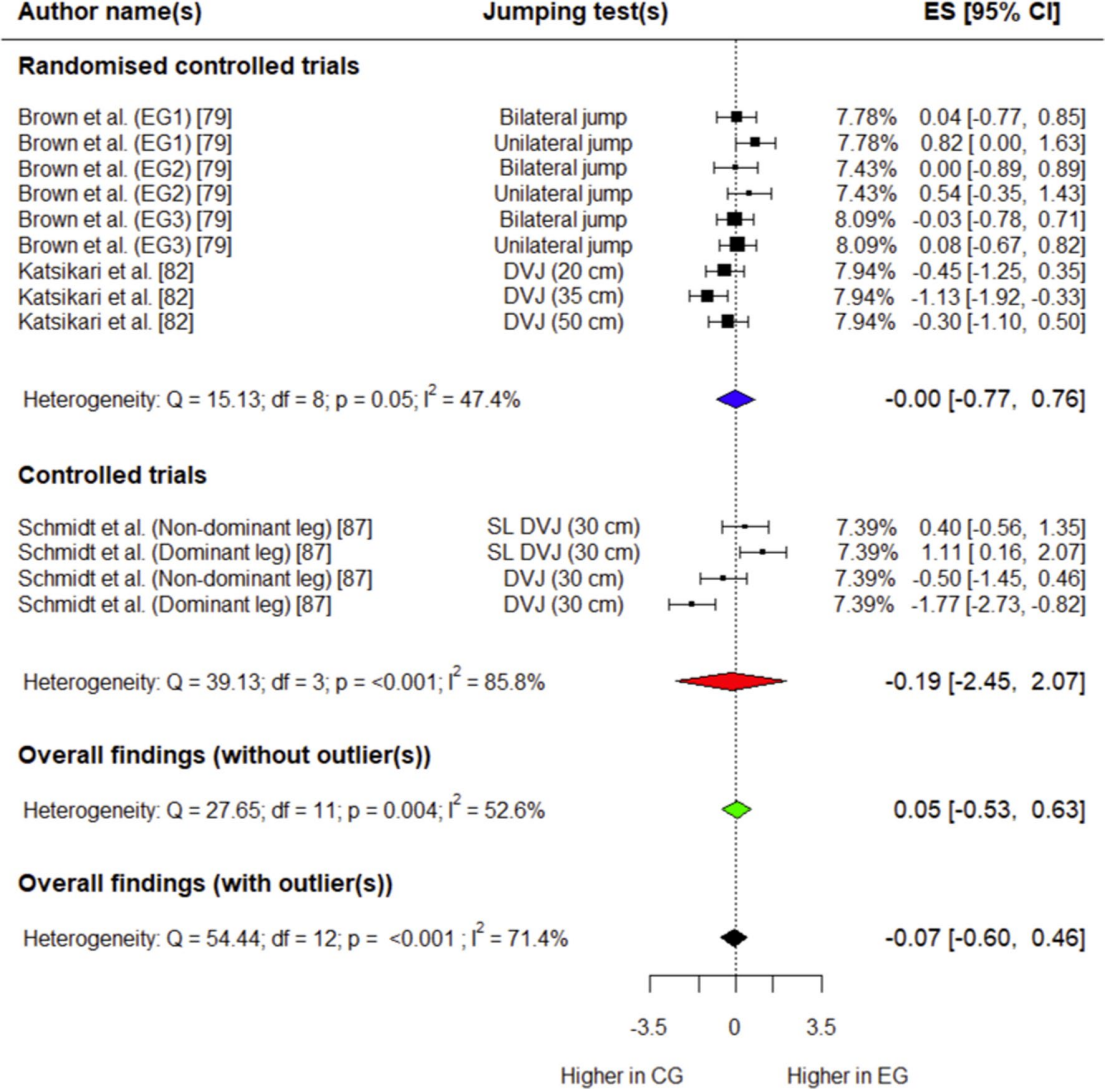
Forest plot showing effects of the training intervention on peak knee flexion moment (95% CI, 95% confidence interval; CG, control group; DVJ, drop vertical jump; EG, experimental group; ES, effect size; SL, single-leg)

Three studies (two RCTs [79, 82] and one CT [87]) involving 86 (54 in experimental group, 32 in control group) participants provided data for this outcome. The experimental group had a trivial, non-significant decrease in peak knee flexion moment compared with the control group (*g* =  − 0.07; 95% CI − 0.60 to 0.46; 95% PI − 2.03 to 1.89; *p* = 0.73) (Fig. 10). Overall heterogeneity was moderate for this variable (*I*^2^ = 71.4%). After influential analysis (Supplementary Fig. S14), the control group had a small, non-significant decrease in peak knee flexion moment compared with the experimental group (*g* = 0.05; 95% CI − 0.53 to 0.63; 95% PI − 1.32 to 1.42; *p* = 0.82) (Fig. 10). Overall heterogeneity was moderate after influential analysis (*I*^2^ = 52.6%).

##### Peak Knee Abduction Moment

**Fig. 11:**
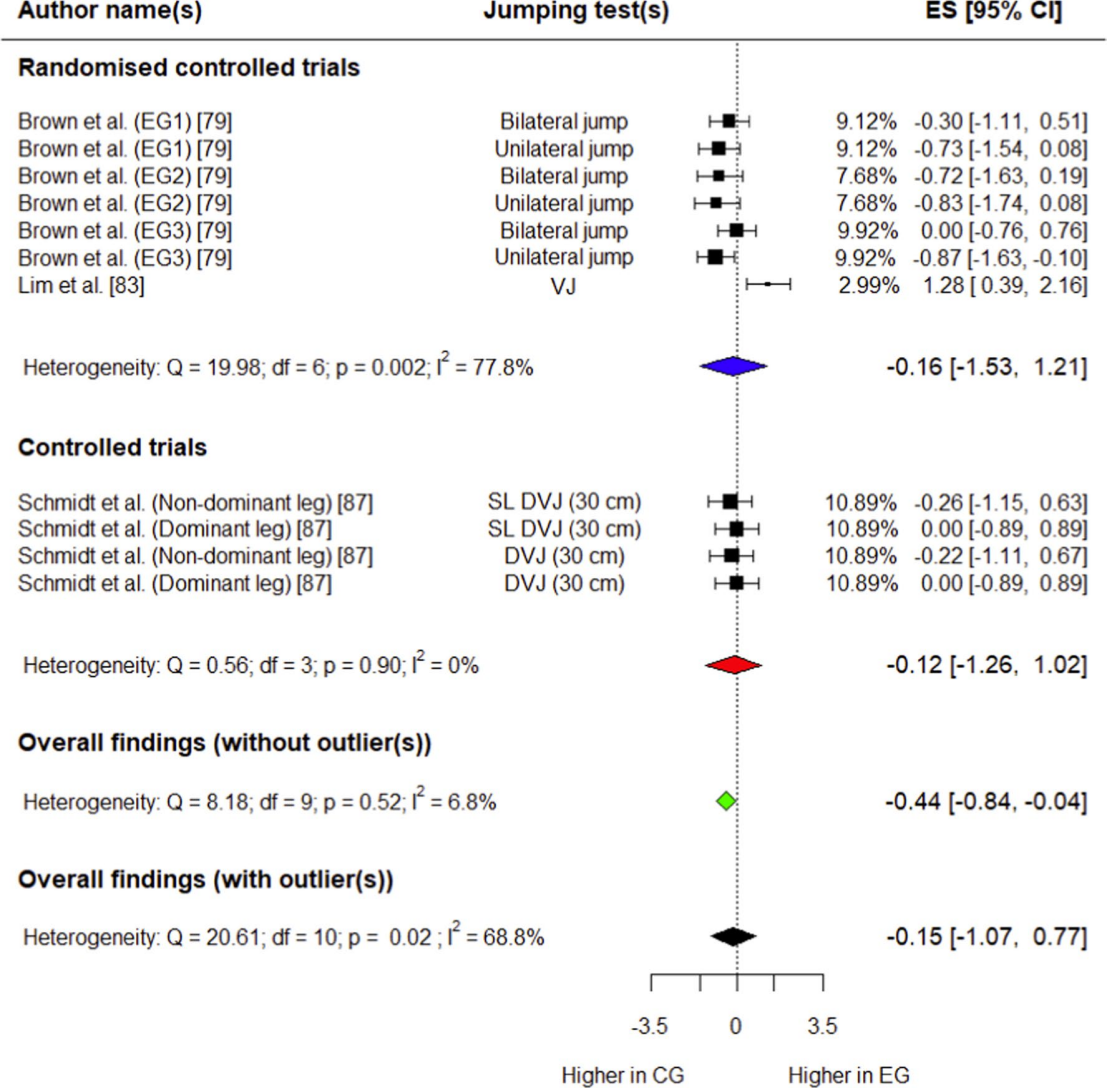
Forest plot showing effects of the training intervention on peak knee abduction moment (95% CI, 95% confidence interval; CG, control group; DVJ, drop vertical jump; EG, experimental group; ES, effect size; SL, single-leg; VJ, vertical jump)

Three studies (two RCTs [79, 83] and one CT [87]) involving 84 (53 in experimental group, 31 in control group) participants provided data for this outcome. The control group had a trivial, non-significant decrease in peak knee abduction moment compared with the experimental group (*g* =  − 0.15; 95% CI − 1.07 to 0.77; 95% PI − 2.16 to 1.86; *p* = 0.68) (Fig. 11). Overall heterogeneity was moderate for this variable (*I*^2^ = 68.8%). After conducting outlier analysis (Supplementary Fig. S15), the control group had a small, non-significant decrease in peak knee abduction moment compared with the experimental group (*p* = 0.08) (Fig. 11). Overall heterogeneity was trivial after influential analysis (*I*^2^ = 6.8%).

Further detailed information regarding the within and between group effect sizes for each kinematic and kinetic variables based on the study design (RCTs and CTs) is provided in Table 4. Funnel plots for each kinematic and kinetic variable are provided in Supplementary Figs. S1–S9. The Cook’s distance plot for the kinematic and kinetic variables for which outliers were detected are provided in Supplementary Figs. S10–S15.

**Table 4:** Overall meta-analytical findings for the kinematic and kinetic variables

Variable	Study design	No. of studies	Effect size	Effect	95% CI	*p*-Value	*I*^2^ (within studies)	*I*^2^ (between studies)	*I*^2^ (overall)	Tau^2^ (within studies)	Tau^3^ (between studies)	95% PI
**Kinematic variables**												
Peak hip adduction angle	RCT	2	0.09	Trivial	− 0.53 to 0.72	0.67	0%	17.6%	17.6%	0	0.18	− 0.76 to 0.95
	CT	2	0.04	Trivial	− 1.54 to 1.61	0.82	0.6%	0%	0.6%	0.03	0	− 1.58 to 1.65
	***Overall findings (no outlier detected)***	***4***	***0.07***	***Trivial***	*** − 0.23 to 0.37***	***0.56***	***0%***	***0%***	***0%***	***0***	***0***	*** − 0.23 to 0.37***
Peak hip flexion angle	RCT	2	0.30	Small	0.04 to 0.56	0.03	0%	0%	0%	0	0	0.04 to 0.56
	CT	1	1.18	Moderate	0 to 2.36	0.05	0%	0%	0	0	0	0 to 2.36
	***Overall findings (without outlier)***	***2***	***0.60***	***Moderate***	*** − 0.06 to 1.27***	***0.06***	***0%***	***15%***	***15%***	***0***	***0.19***	*** − 0.29 to 1.49***
	***Overall findings (with outlier)***	***3***	***0.46***	***Small***	*** − 0.03 to 0.96***	***0.06***	***0%***	***16.4%***	***16.4%***	***0***	***0.19***	*** − 0.25 to 1.18***
Peak knee flexion angle	RCT	5	0.82	Moderate	0.17 to 1.47	0.02	17%	50.9%	67.9%	0.31	0.54	− 0.83 to 2.47
	CT	3	0.08	Trivial	− 0.21 to 0.37	0.36	24.7%	0%	24.7%	0.23	0	− 0.93 to 1.09
	***Overall findings (without outlier)***	***6***	***0.51***	***Small***	*** − 0.05 to 1.07***	***0.07***	***10.8%***	***56.5%***	***67.3%***	***0.24***	***0.54***	*** − 1.00 to 2.02***
	***Overall findings (with outlier)***	***8***	***0.58***	***Small***	***0.11 to 1.05***	***0.02***	***16.1%***	***51%***	***67.1%***	***0.29***	***0.51***	*** − 0.83 to 1.98***
Peak knee abduction angle	RCT	2	0.06	Trivial	− 0.06 to 0.18	0.20	0%	0%	0%	0	0	− 0.06 to 0.18
	CT	4	− 0.35	Small	− 0.85 to 0.15	0.11	57.5%	0%	57.5%	0.44	0	− 1.84 to 1.14
	***Overall findings (without outlier)***	***6***	*** − 0.11***	***Trivial***	*** − 0.34 to 0.12***	***0.31***	***0%***	***0%***	***0%***	***0***	***0***	*** − 0.34 to 0.12***
	***Overall findings (with outlier)***	***6***	*** − 0.17***	***Trivial***	*** − 0.42 to 0.08***	***0.15***	***39.7%***	***0%***	***39.7%***	***0.31***	***0***	*** − 0.95 to 0.61***
Knee abduction angle at IC	RCT	1	0.03	Trivial	− 4.42 to 4.49	0.94	0%	0%	0%	0	0	− 4.42 to 4.49
	CT	2	− 0.09	Trivial	− 0.92 to 0.73	0.67	0%	21.8%	21.8%	0	0.19	− 1.24 to 1.05
	***Overall findings (no outlier detected)***	***3***	*** − 0.05***	***Trivial***	*** − 0.47 to 0.37***	***0.71***	***0%***	***0%***	***0%***	***0***	***0***	*** − 0.47 to 0.37***
Knee flexion ROM	RCT	1	− 0.36	Small	− 0.71 to − 0.00	–	–	–	–	–	–	to
	Non-RCT	2	0.39	Small	− 0.05 to 0.83	0.07	2.1%	2.1%	4.2%	0.04	0.04	− 0.09 to 0.87
	***Overall findings (no outlier detected)***	***3***	***0.22***	***Small***	*** − 0.35 to 0.78***	***0.34***	***34.6%***	***34.6%***	***69.2%***	***0.26***	***0.26***	*** − 0.96 to 1.40***
Knee valgus motion	RCT	1	− 1.72	Large	− 2.31 to − 1.31	–	–	–	–	–	–	–
	CT	2	− 0.59	Small	− 0.81 to − 0.37	0.004	0%	0%	0%	0	0	− 0.81 to − 0.37
	***Overall findings (without outlier)***	***2***	*** − 0.59***	***Small***	*** − 0.81 to − 0.37***	***0.004***	***0%***	***0%***	***0%***	***0***	***0***	*** − 0.81 to − 0.37***
	***Overall findings (with outlier)***	***3***	*** − 0.86***	***Moderate***	*** − 1.55 to − 0.17***	***0.03***	***38.7%***	***38.7%***	***77.4%***	***0.34***	***0.34***	*** − 2.37 to 0.65***
**Kinetic variables**												
Peak knee flexion moment	RCT	2	0.00	No effect	− 0.77 to 0.76	0.99	23.4%	24%	47.4%	0.27	0.28	− 1.46 to 1.45
	CT	1	− 0.19	Trivial	− 2.45 to 2.07	0.81	85.8%	0%	85.8%	1.19	0	− 4.61 to 4.23
	***Overall findings (without outlier)***	***3***	***0.05***	***Trivial***	*** − 0.53 to 0.63***	***0.82***	***48.6%***	***4%***	***52.6%***	***0.42***	***0.12***	*** − 1.32 to 1.42***
	***Overall findings (with outlier)***	***3***	*** − 0.07***	***Trivial***	*** − 0.60 to 0.46***	***0.73***	***71.4%***	***0%***	***71.4%***	***0.68***	***0***	*** − 2.03 to 1.89***
Peak knee abduction moment	RCT	2	− 0.16	Trivial	− 1.53 to 1.21	0.74	12.5%	65.3%	77.8%	0.32	0.73	− 3.03 to 2.71
	CT	1	− 0.12	Trivial	− 1.26 to 1.02	0.76	0%	0%	0%	0	0	− 1.26 to 1.02
	***Overall findings (without outlier)***	***2***	*** − 0.44***	***Small***	*** − 0.84 to − 0.04***	***0.03***	***6.8%***	***0%***	***6.8%***	***0.12***	***0***	*** − 0.99 to 0.10***
	***Overall findings (with outlier)***	***3***	*** − 0.15***	***Trivial***	*** − 1.07 to 0.77***	***0.68***	***4.5%***	***64.3%***	***68.8%***	***0.16***	***0.62***	*** − 2.16 to 1.86***

Abbreviations: CI, confidence interval; CT, controlled trials; IC, initial contact; PI, prediction interval; RCT, randomized controlled trial; ROM, range of motion

## Discussion

Our review aimed to provide a quantitative summary of the effectiveness of various training interventions on jump-landing biomechanics in young females. Of the seven kinematic variables that were analysed in the meta-analysis, training interventions significantly increased peak knee flexion angle and reduced knee valgus motion in the experimental group compared with control group, albeit with small to moderate effects. Effects on other variables (peak hip adduction and flexion angles, peak knee abduction angle, knee abduction angle at IC, knee flexion range of motion) ranged from trivial to moderate. The results indicated that kinetic variables (peak knee flexion moment and peak knee abduction moment) were not significantly altered as a result of various training interventions.

Our analysis showed that youth females achieved small, significant increases in knee flexion angle (*g* = 0.58, *p* = 0.02) during jump-landing tasks following applied training interventions. These findings are in line with previous meta-analysis which have reported improvement in peak knee flexion angles post-training interventions in females [41, 62]. Further, there is evidence in the existing literature that landing with reduced knee flexion can increase the risk of athletes sustaining ACL injuries [31, 32, 89, 90]. For example, injured high-school adolescent female athletes had a peak knee flexion angle that was ~ 10.5° less than uninjured peers [31]. Less knee flexion during landing can result in increased anterior tibial shear load [91], especially in early deceleration phases of movement, which can increase the possibility of athletes sustaining an ACL injury. The interventions in this meta-analysis appeared to induce positive adaptations in youth females, enabling them to land with greater knee flexion. A possible mechanism by which increased knee flexion can protect athletes from ACL injury is by placing the hamstrings in an advantageous position for muscle action [92], which potentially allows them to act as ACL synergists by pulling the tibia posteriorly and thus decreasing anterior tibial force [93], as well as improving energy absorption [94] during landing. Further, Leppanen et al. [32] reported that female athletes landing with higher peak knee flexion moments were at higher risk of sustaining ACL injury. We found a trivial, non-significant reduction in peak knee flexor moments in the training intervention group compared with controls. However, it is currently unclear whether these trivial to small changes in knee flexion angle or moment are sufficient to reduce risk of ACL injury in youth females, especially those at high risk of injury. Therefore, more targeted plyometric or strength training interventions aimed at increasing knee flexion angle/moment should be further explored.

Female athletes tend to land with a straight knee, which indicates greater reliance on the quadriceps muscles [95, 96]. This landing technique can result in decreased hamstring strength and activation relative to the quadriceps muscle [95, 97], which has been reported to be a possible contributing factor to ACL injury [98]. Plyometric training could be considered as a potential solution as this form of training has been found to reduce extension moments [99, 100] and improve muscle strength and neuromuscular control of the knee extension muscles [101], which in turn could reduce the ACL load. The improvement in muscle strength can in turn lead to an increase in the “cushioning” time during the landing phase, leading to greater knee flexion angles and reduced extension moments [102]. Of the eight studies that reported peak knee flexion values, only two studies [79, 82] had incorporated plyometric training as standalone sessions, with other studies combining it with other forms of training (e.g. balance, strength or COD train). Therefore, further research is required in young females to explore the effects of plyometric training on jump-landing biomechanics. A recent review reported that 1–3 sessions of movement/technique training can lead to improvements in knee flexion angles during jump-landing tasks [103]. Therefore, incorporating this performance enhancement technique in the training routine of youth female seems to be useful in increasing the knee flexion angles during jump-landing tasks.

We found moderate, significant reductions in knee valgus motion in the experimental group compared with controls (*g* =  − 0.86, *p* = 0.03). Studies calculated the variable by measuring displacement of patella markers between the frame before initial contact and the maximum medial knee position. Knee valgus is a movement that involves a combination of hip adduction, knee abduction and ankle eversion [31]. Knee valgus is associated with ACL injuries in adolescent female soccer players [104], and interventions targeting this biomechanical variable are deemed important to reduce injury [98]. All three studies [80, 84, 85] reporting the knee valgus motion had components of strength, plyometric and COD/agility training (Supplementary Table S2) as part of their specific injury prevention programme. Also, two studies stated that they focussed on reducing the knee valgus motion as part of the training intervention, although information regarding the cueing technique/instructions for performing exercises was not reported in both studies [84, 85]. Therefore, the multimodal nature combined with the specificity of the training interventions to reduce knee valgus motion could have contributed to the significant improvement of this biomechanical variable. Further, the duration of the training interventions that reported knee valgus motion [80, 84, 85] ranged from 16 to 24 weeks, which may have helped them amass a greater cumulative training dosage compared with other training interventions studies included in our review which lasted between 6 and 10 weeks [5, 65, 66, 79, 81–83, 86–88]. Notably, previous meta-analyses have identified that longer training durations are more beneficial to reduce ACL injuries [105, 106]. Longer-duration interventions inevitably allow participants to be progressively exposed to higher intensities of training, which will likely have driven the moderate, significant reduction in knee valgus motion reported in this meta-analysis.

Surprisingly, knee abduction and knee moment did not differ between experimental and control groups following the training intervention. The type of training intervention administered could be one potential reason for the lack of meaningful difference between groups. Of the six studies included in our analyses, four studies administered the FIFA 11 + and FMARC 11 + injury prevention programmes during warm-up. These exercises are typically performed for a maximum of ~ 20–30 min prior to training, which arguably does not provide enough volume or intensity for individualised training to evoke large, sustained changes in lower limb biomechanics (e.g. knee abduction) akin to those from targeted training programmes with greater training exposures (e.g. 60–90 min [36]). While two studies included in our meta-analysis completed the training intervention in separate dedicated sessions for a duration of ~ 60 min, there were minimal or no improvements in the experimental compared with control group in both studies [79, 87]. However, it should be noted that the frequency of training in the study by Schmidt et al. [87] was only a single session per week, and higher training frequencies (e.g. > 2–3 times/week) are more effective in improving jump-landing biomechanics [50].

In addition to training volume and intensity, exercise selection also may play a role in determining training adaptations [107, 108]. For example, plyometric exercises such as squat jumps, vertical jumps, single-leg forward landing, wall jumps and box jumps were performed as part of the training programmes in four studies [65, 66, 79, 88]. Even though plyometric exercises are considered multiplanar in nature, the exercises used in many studies included in this meta-analysis primarily target motion in the sagittal plane and may not provide sufficient stimulus to the surrounding muscles in the frontal plane that help in stabilising the knee joint, which might explain our findings of significant improvements in pooled peak knee flexion angle but not knee abduction angle and moment, which are primarily frontal plane movements. This could further explain the lack of improvement in knee abduction angle in the Brown et al. [79] study despite the frequency of the training sessions being three times/week. Generally, injury prevention programmes are designed to incorporate a large array of training components (e.g. strength, balance, plyometrics), limiting the depth in any one single training domain [109]. Crossley et al. [46] reported that only 30–67% of injury prevention programmes meet the guidelines (i.e. sets, repetitions, duration and progression in intensity/difficulty) for strength and plyometric/power training in female athletes. Further, a recent scoping review reported that 81.8% of the exercises in ACL injury prevention programmes have a sagittal plane dominance [110]. Therefore, implementation of more targeted programmes or exercises focused on targeting frontal plane kinematics and kinetics may facilitate improvements in jump-landing biomechanics. Further, it is recommended that the athletes should be screened for specific risk factors prior to the start of a training intervention. For instance, a 7-week injury prevention programme was effective in reducing the knee abduction moment in young females who were in the ‘high-risk’ category (39.9–34.6 Nm), whilst the decrease in knee abduction moment was non-significant in athletes in the ‘low risk’ category (14.5–14.8 Nm versus 14.7–17.5 Nm) [111]. This further indicates that the training interventions might not make much difference if the participants do not show any signs of that particular deficit being present. Therefore, practitioners should consider developing and implementing individualised interventions based on the needs of an athlete rather than prescribing a generic program [41].

There are certain limitations within the current study that warrant consideration. Firstly, we pooled results from various study designs in our analysis, which could have influenced the overall effect estimates. However, we also provided pooled effect sizes based on the study design (i.e. RCT versus CT) for each kinematic and kinetic variable to counter this limitation. Secondly, differences in training protocols (i.e. duration, frequency, volume, intensity) of the interventions, within-group effect sizes which could lead to larger type 1 errors, data collection methodologies (i.e. two- versus three-dimensional motion capture systems) and various measurement units for the reported kinetic variables (N, Nm or BW) could have introduced heterogeneity in our findings. However, to limit this effect, we used a multi-level, meta-analytical model and analysed each variable without outliers (if detected) to provide a robust estimate for each variable reported.

## Future Research Directions

The certainty of evidence for all kinematic and kinetic variables was found to be very low. Our analysis for each variable involved a combination of RCTs and CTs, and the level of evidence was downgraded at the very beginning in accordance with the GRADE criteria. This could be the primary reason for the low level of evidence. As RCTs are considered to provide the highest level of scientific evidence [112], further studies employing this study design need to be conducted in youth females. Also, the domains were primarily downgraded for risk of bias and imprecision given that the total sample size was less than *n* = 800. Studies with small sample size are a common problem in sport science [113], which could lead to inflated type 1 errors, low statistical power and biased results [114–118], and thus it is recommended that authors in biomechanical research perform sample size estimations a priori [114, 119]. Notably, only 4 [79, 81, 85, 86] of the 13 studies (i.e. 30%) included in our review reported sample size/power analysis. Therefore, more high-quality studies (RCTs) with larger sample sizes are required to acquire a better understanding of how training interventions influence jump-landing biomechanics in young females.

The hip and ankle joints influence knee biomechanics during the deceleration phase of jump-landing tasks [14, 120–125]. However, from the studies included in this review, there was insufficient data to pool variables at these two joints and conduct a meta-analysis. In addition to examining the influence of training interventions on knee biomechanics during jump-landing tasks, future research should also explore how changes in kinetics and kinematics at the hip and ankle contribute to ACL injury risk in youth females.

Data indicate that ACL injuries in female athletes increase around the time of puberty [15, 126, 127], and several training interventions have been developed to influence the landing biomechanics in females [36, 37, 128–137]. These changes related to ACL injury risk start developing during early puberty [16, 19, 20, 138, 139], and previous meta-analytical data have indicated that there exists an age-related association between injury prevention programme implementation and subsequent reduction in ACL incidence, with early engagement during early adolescence most effective [140]. However, there is currently little evidence on the magnitude of change across various kinetic and kinematic ACL risk factors as a result of training interventions at different stages of maturation in females. Given the limitations of the existing data, we were unable to perform a moderating analysis exploring the influence of maturity status on biomechanical adaptations to training interventions. Therefore, it is recommended that future research should aim to account for the mediating role of interactions between growth, maturation and training.

Finally, the studies included in our meta-analysis focussed on discrete time points such as initial contact or peak values for joint angles and moments. Although this method provides valuable information regarding the changes in kinematic and kinetic variables while landing from jumping tasks pre- and post-training intervention, the reliance on single time point variables fails to provide insight into continuous time series data, which may provide a more granular level of analysis [141, 142]. Such an approach might also limit our understanding on how the hip, knee and ankle joints behave in response to the training interventions throughout the eccentric phase of jump landing. Therefore, it is recommended that future studies should consider using more nuanced techniques such as statistical parametric mapping (SPM), principal component analysis (PCA) or factor analysis in order to overcome this issue.

## Conclusions

Overall, training interventions aimed at reducing the risk of ACL injury in young females have significantly increased peak knee flexion angle and reduced knee valgus motion in the experimental groups compared with controls. The effects on all other kinematic and kinetic variables were non-significant and trivial to moderate in magnitude. Further research is needed to explore the efficacy of more targeted long-term training interventions aimed at improving multi-planar kinetic and kinematic ACL injury risk factors in young females. Whilst we were unable to conduct a moderator analysis, previous meta-analyses have indicated that training durations of > 8 weeks with a training frequency ranging between 2 and 3 sessions/week provide an effective training stimulus [143, 144]. In order for the training programmes to be more effective, practitioners are encouraged to screen athletes for neuromuscular deficits during jump-landing tasks and develop individualised training programmes accordingly. Previous research findings [111, 145, 146] indicate that this approach could further improve jump-landing biomechanics and in turn help to reduce the incidence of ACL injuries in young females.

## Supplementary Information

Below is the link to the electronic supplementary material.

Supplementary file1 (DOCX 20 KB)

Supplementary file2 (DOCX 296 KB)

Supplementary file3 (DOCX 42 KB)

## Declarations

### Data Availability

All data and material reported in this systematic review are from peer-reviewed publications and have been included in this study as tables, figures and supplementary material. Any other data requirement can be directed to the corresponding author upon reasonable request.
