# Supplementary material for: Influence of Neuromuscular Training Interventions on Jump-Landing Biomechanics and Implications for ACL Injuries in Youth Females: A Systematic Review and Meta-analysis
Source: Sports Med. 2025 Apr 17;55(5):1265–92. doi: 10.1007/s40279-025-02190-w (PMC12106595; doi:10.1007/s40279-025-02190-w)
Supplement: Supplementary file 1 — Supplementary file1 (DOCX 20 KB) [file 40279_2025_2190_MOESM1_ESM.docx]

**Supplementary Material Appendix S1**

**Influence of neuromuscular training interventions on jump-landing biomechanics and implications for ACL injuries in youth females: a systematic review and meta-analysis**

**Short title:** Meta-analysis of training intervention impacts on jump landing biomechanics in youth females

**Authors:**

Akhilesh Kumar Ramachandran^1^; Jason S. Pedley^1^; Sylvia Moeskops^1^; Jon L. Oliver^1,2^; Gregory D. Myer^1,4,5,6,7,8;^ Hung-I Hsiao^4,5,6^; Rhodri S. Lloyd^1,2,3^

**Affiliations:**

1. Youth Physical Development Centre, Cardiff School of Sport and Health Sciences, Cardiff Metropolitan University, Cardiff, UK
2. Sport Performance Research Institute, New Zealand (SPRINZ), AUT University, Auckland, New Zealand
3. Centre for Sport Science and Human Performance, Waikato Institute of Technology, Hamilton, New Zealand
4. Emory Sports Performance And Research Center (SPARC), Flowery Branch, GA, USA
5. Emory Sports Medicine Center, Atlanta, GA, USA
6. Department of Orthopaedics, Emory University School of Medicine, Atlanta, GA, USA
7. Wallace H. Coulter Department of Biomedical Engineering, Georgia Institute of Technology & Emory University, Atlanta, GA, USA
8. The Micheli Center for Sports Injury Prevention, Waltham, MA, USA

**Correspondence**

Name: Akhilesh Kumar Ramachandran

Address: Youth Physical Development Centre, Cardiff School of Sport and Health Sciences, Cardiff Metropolitan University, Cyncoed Campus, Cyncoed Road, Cardiff, CF23 6XD, United Kingdom

Email: [aramachandran@cardiffmet.ac.uk](mailto:aramachandran@cardiffmet.ac.uk)

1. **PUBMED (Medline)**

("growth" OR "Matur*" OR "Pubert*" OR "Young" OR “Youth” OR “Adolescent” OR “Adolescence” OR “Tanner stage*” OR “Sex” OR “Female” OR “Girl*” OR “Women”) AND ("Biomechanic*" OR "Mechanic*" OR "kinematic*" OR "kinetic*" OR “Neuromuscular” OR “electromyography” OR “EMG”) AND (“jump landing” OR “jump-landing” OR “jumping” OR “jump test*” OR “jump” OR “jumps” OR “land” OR “landing” OR “landing task*” OR “ landing activit*” OR “landing mechanic*”) AND ("intervention*" OR "training" OR “protocol*” OR "program*" OR "injury prevention program*" OR “prevention” OR “IPP” OR “exercise*” OR “warm-up” OR “warm up”) AND (“trunk” OR “knee” OR “ankle” OR “hip” OR “leg” OR “lower extremet*” OR “limb*” OR “lower limb*” OR “lower-limb*”) = **2,325 results**

1. **SCOPUS**

TIT-ABS-KEY ("growth" OR "Matur*" OR "Pubert*" OR "Young" OR “Youth” OR “Adolescent” OR “Adolescence” OR “Tanner stage*” OR “Sex” OR “Female” OR “Girl*” OR “Women”) AND TIT-ABS-KEY ("Biomechanic*" OR "Mechanic*" OR "kinematic*" OR "kinetic*" OR “Neuromuscular” OR “electromyography” OR “EMG”) AND TIT-ABS-KEY (“jump landing” OR “jump-landing” OR “jumping” OR “jump test*” OR “jump” OR “jumps” OR “land” OR “landing” OR “landing task*” OR “ landing activit*” OR “landing mechanic*”) AND TIT-ABS-KEY ("intervention*" OR "training" OR “protocol*” OR "program*" OR "injury prevention program*" OR “prevention” OR “IPP” OR “exercise*” OR “warm-up” OR “warm up”) AND TIT-ABS-KEY (“trunk” OR “knee” OR “ankle” OR “hip” OR “leg” OR “lower extremet*” OR “limb*” OR “lower limb*” OR “lower-limb*”) = **2,661 results**

1. **SPORTDiscus**

("growth" OR "Matur*" OR "Pubert*" OR "Young" OR “Youth” OR “Adolescent” OR “Adolescence” OR “Tanner stage*” OR “Sex” OR “Female” OR “Girl*” OR “Women”) AND ("Biomechanic*" OR "Mechanic*" OR "kinematic*" OR "kinetic*" OR “Neuromuscular” OR “electromyography” OR “EMG”) AND (“jump landing” OR “jump-landing” OR “jumping” OR “jump test*” OR “jump” OR “jumps” OR “land” OR “landing” OR “landing task*” OR “ landing activit*” OR “landing mechanic*”) AND ("intervention*" OR "training" OR “protocol*” OR "program*" OR "injury prevention program*" OR “prevention” OR “IPP” OR “exercise*” OR “warm-up” OR “warm up”) AND (“trunk” OR “knee” OR “ankle” OR “hip” OR “leg” OR “lower extremet*” OR “limb*” OR “lower limb*” OR “lower-limb*”) = **1,406 results**

1. **EMBASE**

("growth" OR "Matur*" OR "Pubert*" OR "Young" OR “Youth” OR “Adolescent” OR “Adolescence” OR “Tanner stage*” OR “Sex” OR “Female” OR “Girl*” OR “Women”) AND ("Biomechanic*" OR "Mechanic*" OR "kinematic*" OR "kinetic*" OR “Neuromuscular” OR “electromyography” OR “EMG”) AND (“jump landing” OR “jump-landing” OR “jumping” OR “jump test*” OR “jump” OR “jumps” OR “land” OR “landing” OR “landing task*” OR “ landing activit*” OR “landing mechanic*”) AND ("intervention*" OR "training" OR “protocol*” OR "program*" OR "injury prevention program*" OR “prevention” OR “IPP” OR “exercise*” OR “warm-up” OR “warm up”) AND (“trunk” OR “knee” OR “ankle” OR “hip” OR “leg” OR “lower extremet*” OR “limb*” OR “lower limb*” OR “lower-limb*”) = **2,281 results**
