# Supplementary material for: Influence of Neuromuscular Training Interventions on Jump-Landing Biomechanics and Implications for ACL Injuries in Youth Females: A Systematic Review and Meta-analysis
Source: Sports Med. 2025 Apr 17;55(5):1265–92. doi: 10.1007/s40279-025-02190-w (PMC12106595; doi:10.1007/s40279-025-02190-w)

**Supplementary Material Figures**

**Influence of neuromuscular training interventions on jump-landing biomechanics and implications for ACL injuries in youth females: a systematic review and meta-analysis**

**Short title:** Meta-analysis of training intervention impacts on jump landing biomechanics in youth females

**Authors:**

Akhilesh Kumar Ramachandran^1^; Jason S. Pedley^1^; Sylvia Moeskops^1^; Jon L. Oliver^1,2^; Gregory D. Myer^1,4,5,6,7,8;^ Hung-I Hsiao^4,5,6^; Rhodri S. Lloyd^1,2,3^

**Affiliations:**

1. Youth Physical Development Centre, Cardiff School of Sport and Health Sciences, Cardiff Metropolitan University, Cardiff, UK
2. Sport Performance Research Institute, New Zealand (SPRINZ), AUT University, Auckland, New Zealand
3. Centre for Sport Science and Human Performance, Waikato Institute of Technology, Hamilton, New Zealand
4. Emory Sports Performance And Research Center (SPARC), Flowery Branch, GA, USA
5. Emory Sports Medicine Center, Atlanta, GA, USA
6. Department of Orthopaedics, Emory University School of Medicine, Atlanta, GA, USA
7. Wallace H. Coulter Department of Biomedical Engineering, Georgia Institute of Technology & Emory University, Atlanta, GA, USA
8. The Micheli Center for Sports Injury Prevention, Waltham, MA, USA

**Correspondence**

Name: Akhilesh Kumar Ramachandran

Address: Youth Physical Development Centre, Cardiff School of Sport and Health Sciences, Cardiff Metropolitan University, Cyncoed Campus, Cyncoed Road, Cardiff, CF23 6XD, United Kingdom

Email: [aramachandran@cardiffmet.ac.uk](mailto:aramachandran@cardiffmet.ac.uk)

**Fig. S1** Funnel plot for peak hip adduction angle


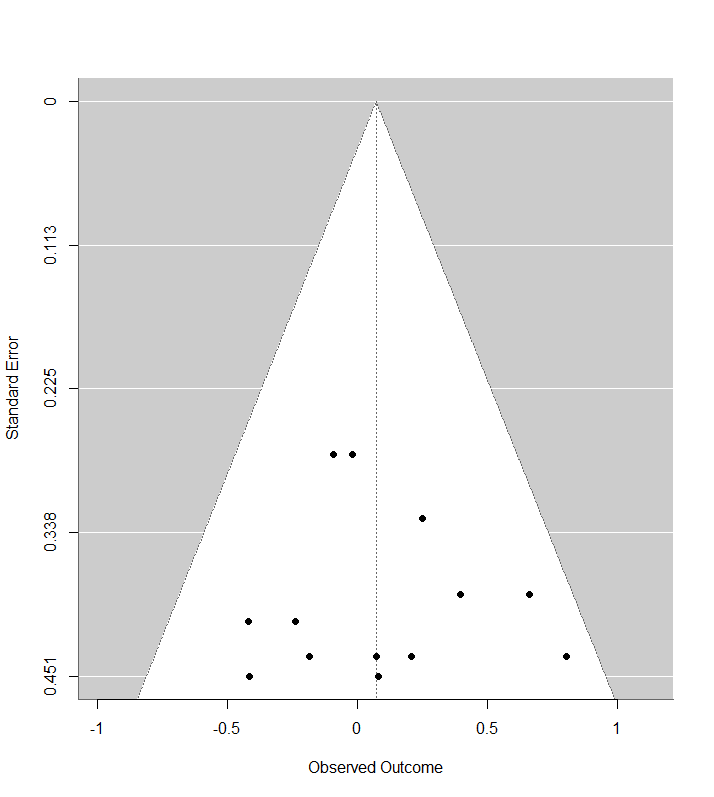


**Fig. S2** Funnel plot for peak hip flexion angle


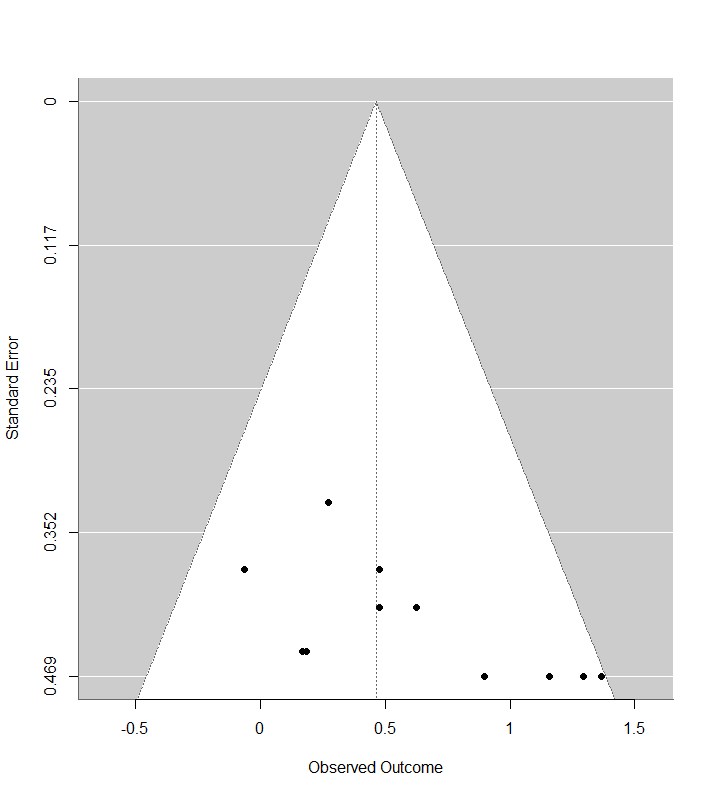


**Fig. S3** Funnel plot for peak knee abduction angle


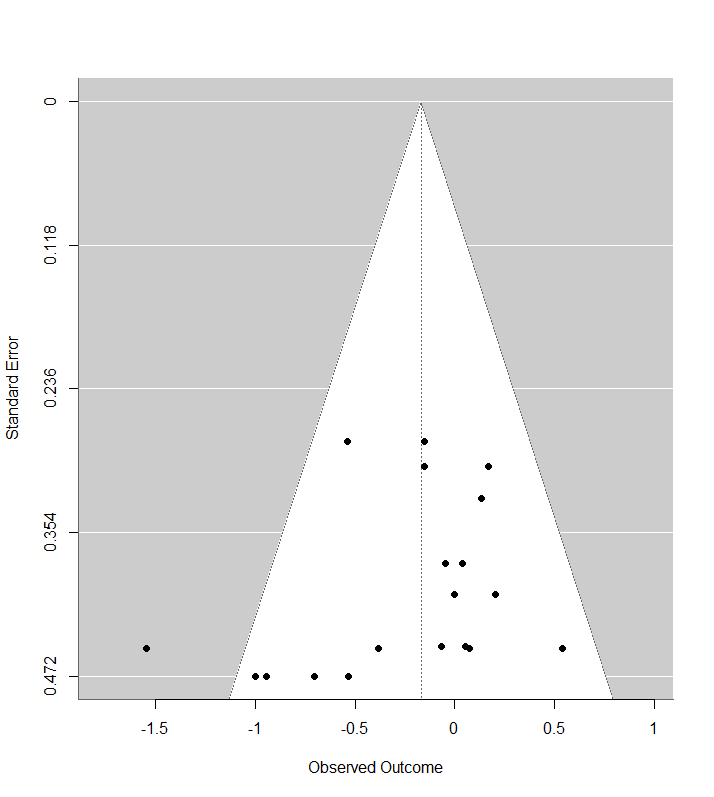


**Fig. S4** Funnel plot for knee abduction angle at initial contact


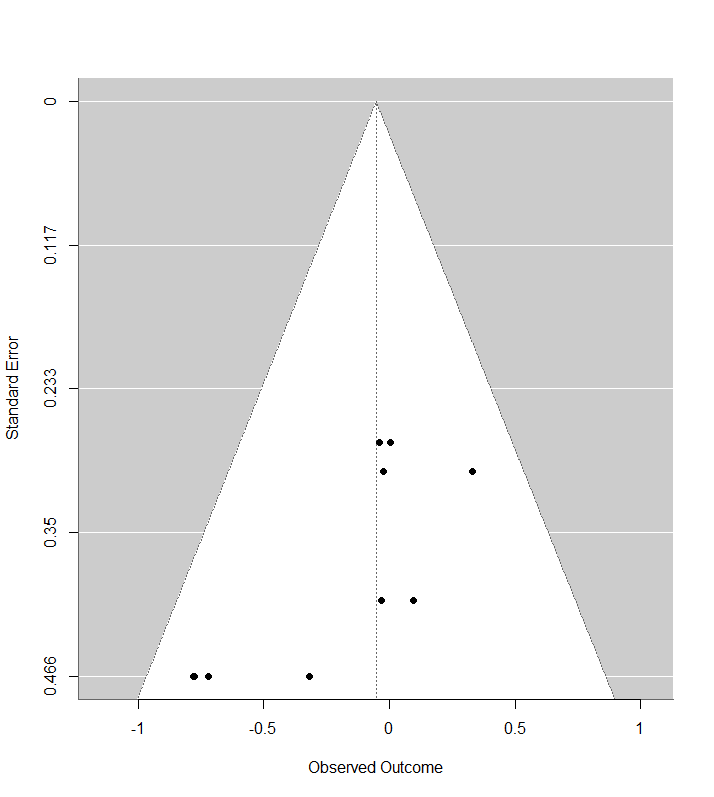


**Fig. S5** Funnel plot for peak knee flexion angle


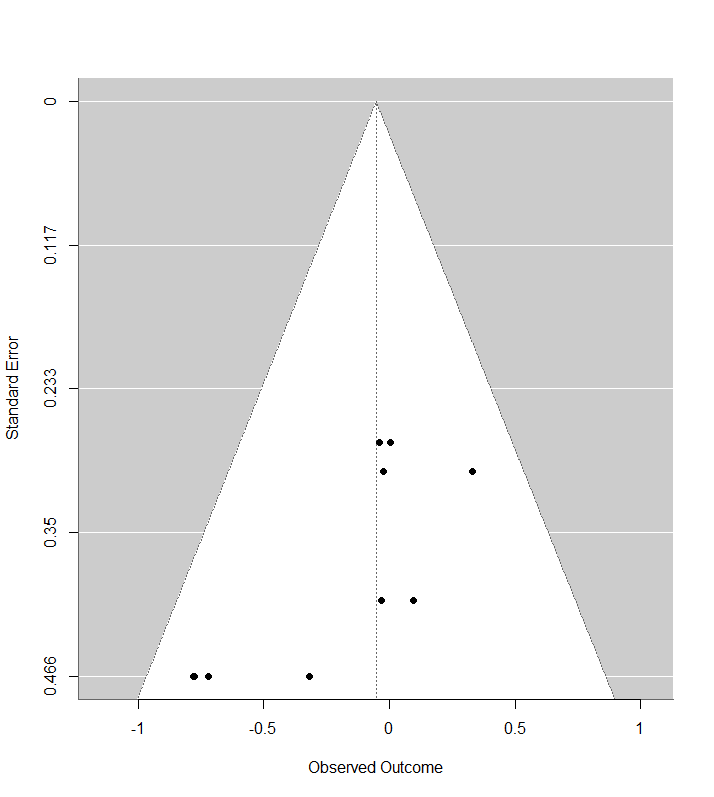


**Fig. S6** Funnel plot for knee flexion range of motion


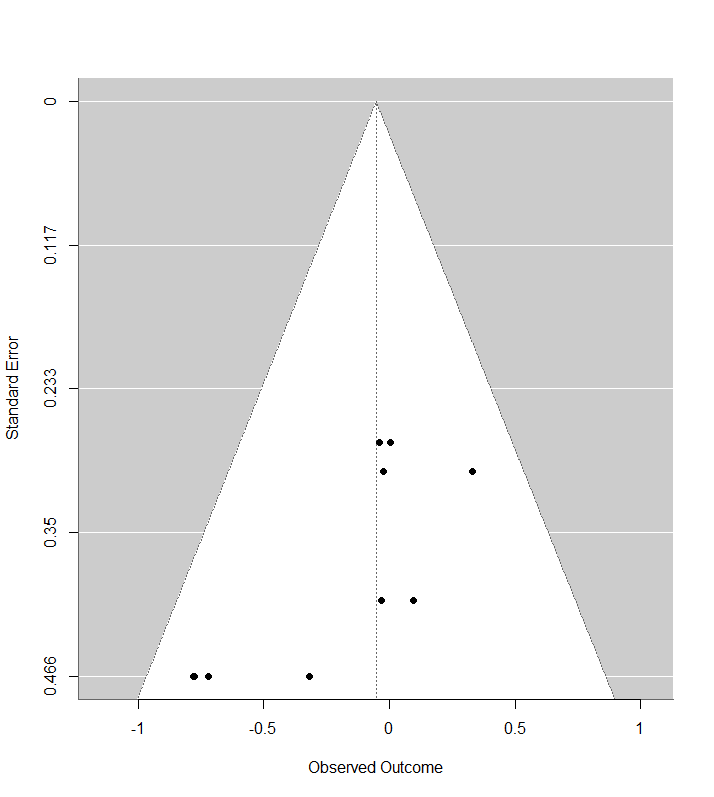


**Fig. S7** Funnel plot for knee valgus motion


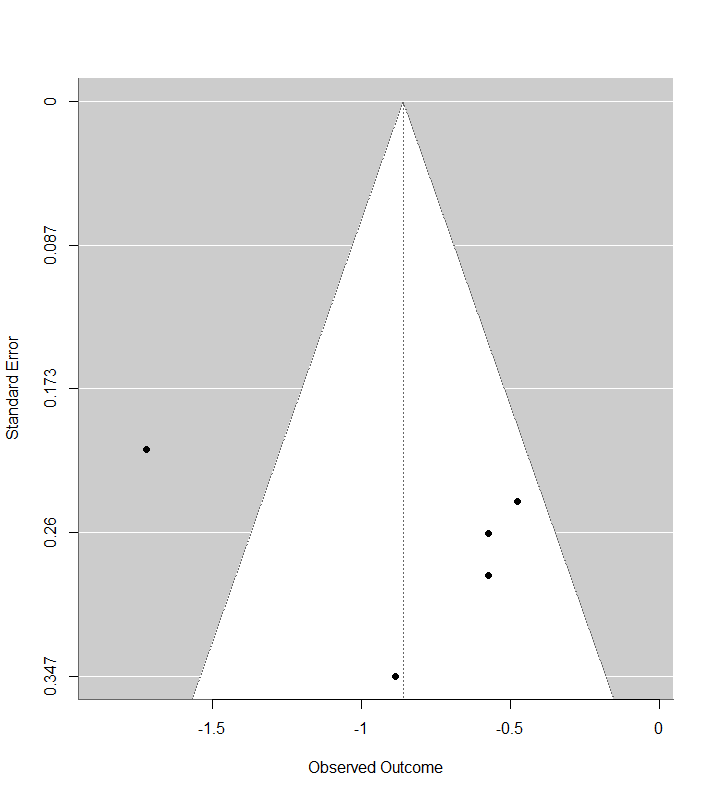


**Fig. S8** Funnel plot for peak knee flexion moment


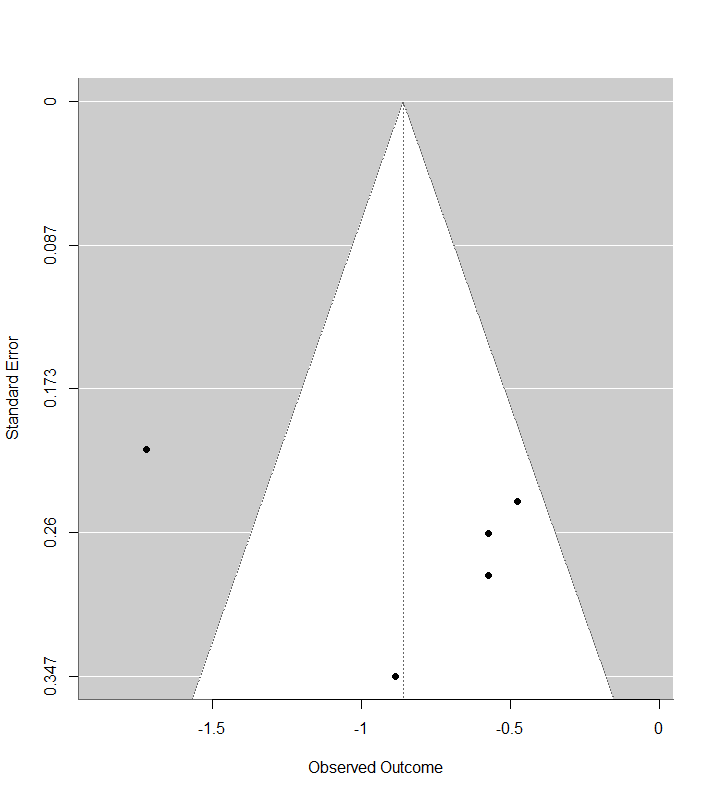


**Fig. S9** Funnel plot for peak knee abduction moment


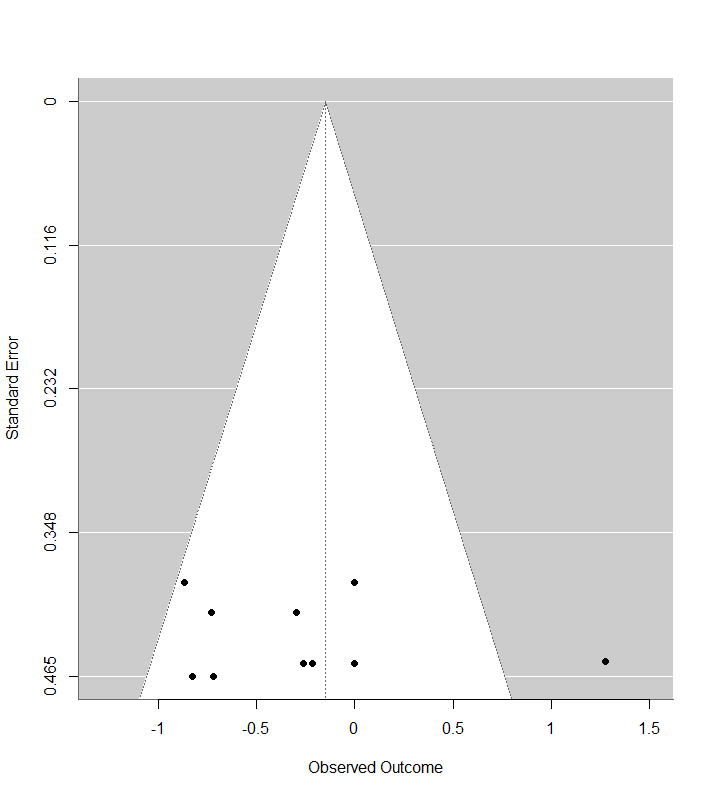


**Fig. S10** Cook’s distance for peak hip flexion angle with outlier(s)


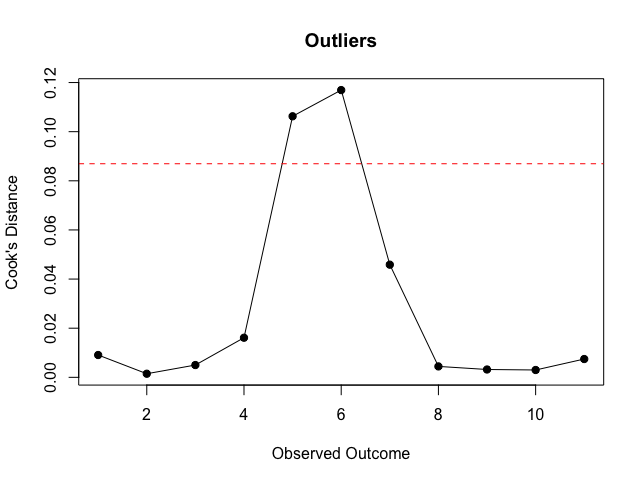


**Fig. S11** Cook’s distance for peak knee flexion angle with outlier(s)


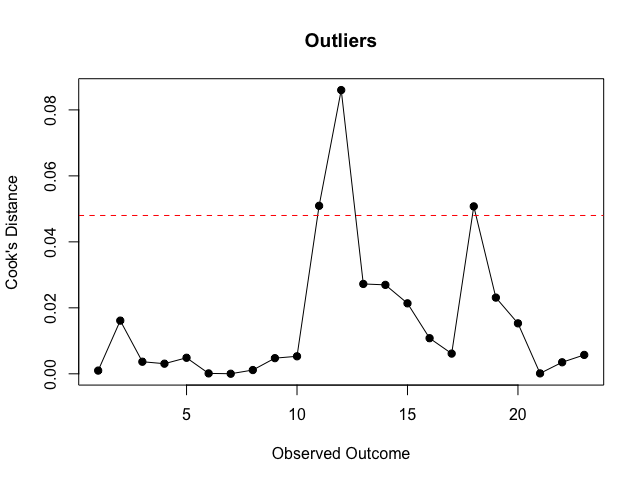


**Fig. S12** Cook’s distance for peak knee abduction angle with outlier(s)


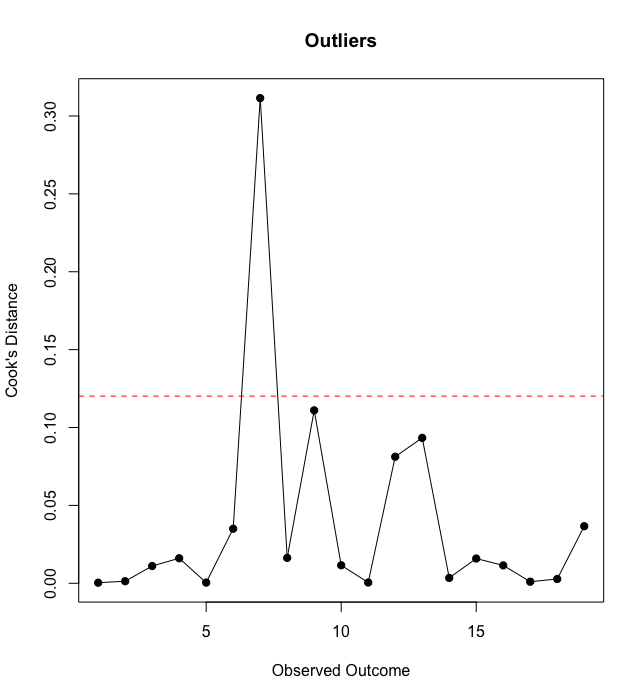


**Fig. S13** Cook’s distance for knee valgus motion with outlier(s)


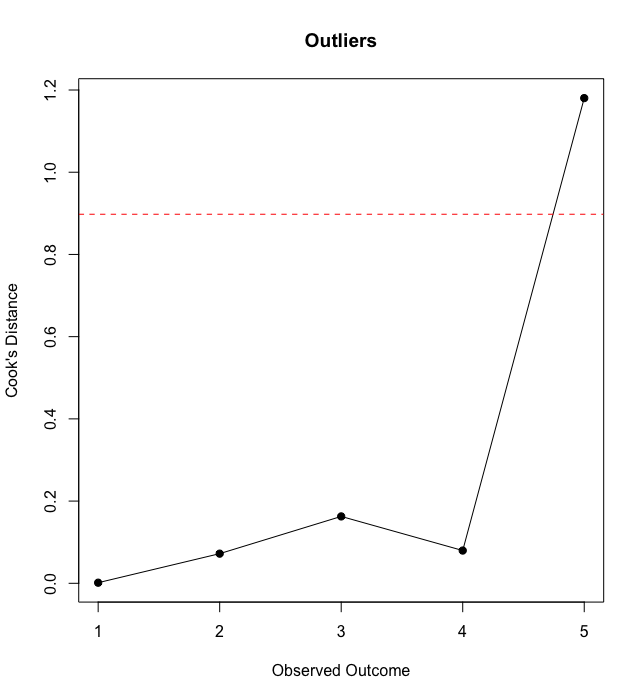


**Fig. S14** Cook’s distance for peak knee flexion moment with outlier(s)


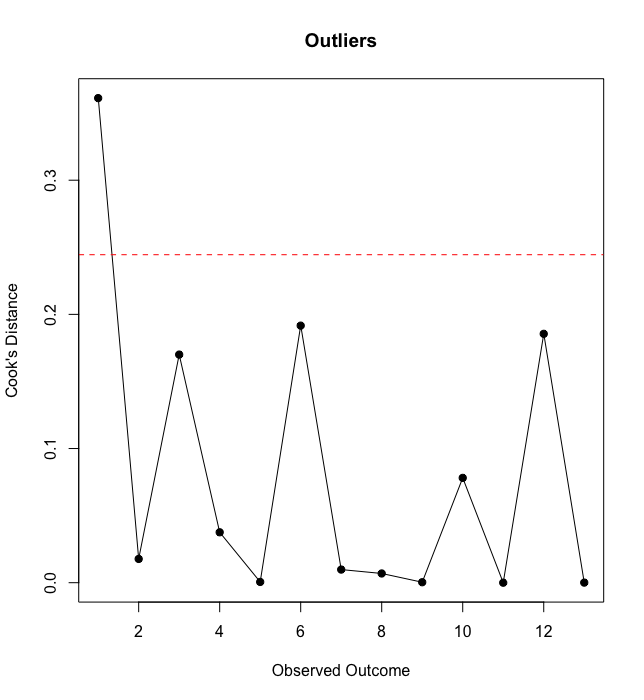


**Fig. S15** Cook’s distance for peak knee abduction moment with outlier(s)


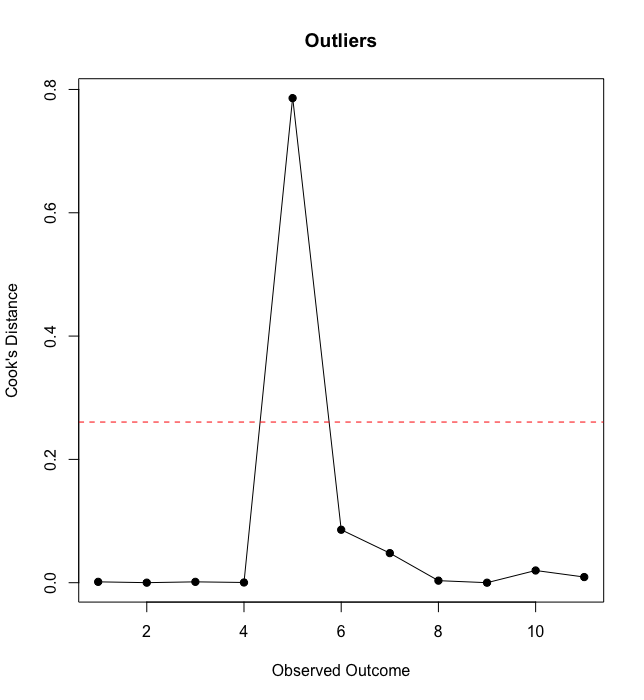

Supplement: Supplementary file 2 — Supplementary file2 (DOCX 296 KB) [file 40279_2025_2190_MOESM2_ESM.docx]
