# Supplementary material for: Influence of Neuromuscular Training Interventions on Jump-Landing Biomechanics and Implications for ACL Injuries in Youth Females: A Systematic Review and Meta-analysis
Source: Sports Med. 2025 Apr 17;55(5):1265–92. doi: 10.1007/s40279-025-02190-w (PMC12106595; doi:10.1007/s40279-025-02190-w)
Supplement: Supplementary file 3 — Supplementary file3 (DOCX 42 KB) [file 40279_2025_2190_MOESM3_ESM.docx]

**Supplementary Material Tables**

**Influence of neuromuscular training interventions on jump-landing biomechanics and implications for ACL injuries in youth females: a systematic review and meta-analysis**

**Short title:** Meta-analysis of training intervention impacts on jump landing biomechanics in youth females

**Authors:**

Akhilesh Kumar Ramachandran^1^; Jason S. Pedley^1^; Sylvia Moeskops^1^; Jon L. Oliver^1,2^; Gregory D. Myer^1,4,5,6,7,8;^ Hung-I Hsiao^4,5,6^; Rhodri S. Lloyd^1,2,3^

**Affiliations:**

1. Youth Physical Development Centre, Cardiff School of Sport and Health Sciences, Cardiff Metropolitan University, Cardiff, UK
2. Sport Performance Research Institute, New Zealand (SPRINZ), AUT University, Auckland, New Zealand
3. Centre for Sport Science and Human Performance, Waikato Institute of Technology, Hamilton, New Zealand
4. Emory Sports Performance And Research Center (SPARC), Flowery Branch, GA, USA
5. Emory Sports Medicine Center, Atlanta, GA, USA
6. Department of Orthopaedics, Emory University School of Medicine, Atlanta, GA, USA
7. Wallace H. Coulter Department of Biomedical Engineering, Georgia Institute of Technology & Emory University, Atlanta, GA, USA
8. The Micheli Center for Sports Injury Prevention, Waltham, MA, USA

**Correspondence**

Name: Akhilesh Kumar Ramachandran

Address: Youth Physical Development Centre, Cardiff School of Sport and Health Sciences, Cardiff Metropolitan University, Cyncoed Campus, Cyncoed Road, Cardiff, CF23 6XD, United Kingdom

Email: [aramachandran@cardiffmet.ac.uk](mailto:aramachandran@cardiffmet.ac.uk)

| **Supplementary Table S1:** GRADE evidence for kinematic and kinetic variables | | | | | | |
| --- | --- | --- | --- | --- | --- | --- |
| **Outcome** | **Risk of bias** | **Inconsistency** | **Indirectness** | **Imprecision** | **Publication bias** | **Certainty of evidence** |
| **Kinematic variables** | | | | | | |
| Peak hip adduction | Serious^a^ | Not serious | Not serious | Serious^b^ | Not serious | ⨁◯◯◯ Very low |
| Peak hip flexion | Serious^a^ | Not serious | Not serious | Serious^b^ | Not serious | ⨁◯◯◯ Very low |
| Peak knee flexion | Serious^a^ | Serious^c^ | Not serious | Serious^b^ | Not serious | ⨁◯◯◯ Very low |
| Peak knee abduction | Serious^a^ | Not serious | Not serious | Serious^b^ | Not serious | ⨁◯◯◯ Very low |
| Knee abduction at initial contact | Serious^a^ | Not serious | Not serious | Serious^b^ | Not serious | ⨁◯◯◯ Very low |
| Knee flexion range of motion | Serious^a^ | Serious^c^ | Not serious | Serious^b^ | Not serious | ⨁◯◯◯ Very low |
| Knee valgus motion | Serious^a^ | Serious^c^ | Not serious | Serious^b^ | Not serious | ⨁◯◯◯ Very low |
| **Kinetic variables** | | | | | | |
| Peak knee flexion moment | Serious^a^ | Serious^c^ | Not serious | Serious^b^ | Not serious | ⨁◯◯◯ Very low |
| Peak abduction moment | Serious^a^ | Serious^c^ | Not serious | Serious^b^ | Not serious | ⨁◯◯◯ Very low |
| Vertical ground reaction force | Serious^a^ | Serious^c^ | Not serious | Serious^b^ | Not serious | ⨁◯◯◯ Very low |

^a^Downgraded due to study reporting the outcome classified as having high risk of bias or some concerns.

^b^Downgraded due to sample size <800.

^c^Downgraded due to I^2^ > 50%.

| **Supplementary Table S2:** Details of the exercises performed in each training intervention group in included studies | | | |
| --- | --- | --- | --- |
| **Study** | **Training intervention** | **Time of exercise administration** | **Exercises administered** |
| Brown et al. [79] | ***EG1:*** Injury prevention training programme (plyometric + core + balance + strength + speed training)  ***EG2:*** Core + balance training  ***EG3:*** Plyometric training | Separate session | ***EG1:*** SWISS crunches; SWISS lateral crunch; SWISS ball lift; SWISS side ball lift; SWISS scissor ball rotation; SWISS seated ball catch; SWISS superman; broad jump—stick hold; BOSU step-up squat; BOSU lateral step-up squat; BOSU balance; BOSU balance—single leg; BOSU squat; Russian twist—ball; BOSU balance knees ball catch; BOSU balance single leg eyes closed; BOSU lateral hop; BOSU squat; BOSU 180 jump—stick and hold; single-leg hop; SWISS crunch—feet on ball; SWISS side ball lift; SWISS scissors; BOSU (f) balance knees—ball catch; BOSU (f) balance single—eyes closed; BOSU (f) squat; broad jump—stick, hold; wall jumps; squat jumps; 180 jumps; bounding; front/back jumps; side/side jumps; broad jumps; triple broad—vertical; scissor jumps; hop, hop, hop, and stick; box jumps; tuck jumps; broad jumps—stick; crossover hop, hop, hop, and stick; 180 jumps—ball catch; box drops; 180 jumps—speed; triple broad—vert; X-hops; depth jumps; box-depth-180-box-depth vertical; SWISS back wall squat; SWISS push-up; lunge—ball twist; band stand pull; calf raise; band stand press; figure 8—ball; squat and throw—ball; push-up—ball; figure 8; jog to sprint; march—light; skipping—medium; run; run—light; run—medium; run—100%; backward run; run—90%; run—heavy.  ***EG2:*** SWISS crunches; SWISS lateral crunch; SWISS ball lift; SWISS side ball lift; SWISS scissor ball rotation; SWISS seated ball catch; SWISS superman; broad jump—stick hold; BOSU step-up squat; BOSU lateral step-up squat; BOSU balance; BOSU balance—single leg; BOSU squat; Russian twist—ball; BOSU balance knees ball catch; BOSU balance single-leg eyes closed; BOSU lateral hop; BOSU squat; BOSU 180 jump—stick and hold; single-leg hop; SWISS crunch—feet on ball; SWISS side ball lift; SWISS scissors; BOSU (f) balance knees—ball catch; BOSU (f) balance single—eyes closed; BOSU (f) squat; broad jump—stick, hold  ***EG3:*** Wall jumps; squat jumps; 180 jumps; bounding; front/back jumps; side/side jumps; broad jumps; triple broad—vertical; scissor jumps; hop, hop, hop, and stick; box jumps; tuck jumps; broad jumps—stick; crossover hop, hop, hop, and stick; 180 jumps—ball catch; box drops; 180 jumps—speed; triple broad—vert; X-hops; depth jumps; box-depth-180-box-depth vertical  ***CG:*** Regular training routine |
| De Ste Croix et al. [80] | Injury prevention training programme  (dynamic warm-up + dynamic flexibility + plyometrics + speed/COD) | Warm-up | ***EG:*** Jog forward; jog backward; low-level skip; high knees out and back; side step out and back; carioca out and back; cross step out and back; rotating side step; L1, static crawls; L2, spiderman crawls; inchworm; L1, static lunge; L2, walking lunges; walking quadriceps stretch; lateral lunge; hamstring starter stretch; L1, arabesque double leg; L2, split stance; L3, single leg; Nordic hamstrings; quick-fire hamstrings; pogos; ankling; L1, squat jumps; L2, countermovement jumps; L3, tuck jumps; alternate step and hold; L1, single-legged hop and hold; L2, single-legged repeated hops; 5 m to 3-step deceleration; 10 m, stop, back pedal 5 m; 15 m, back pedal 5 m, sprint 10 m, and decelerate 10 m, side step, 10 m; 5-m side step out and back to 10-m sprint; 10 m, diagonal cut step, 10 m  ***CG:*** Performed normal warm-up routine |
| Hopper et al. [81] | Injury prevention training programme  (dynamic warm-up + plyometric + strength training) | Separate session | ***EG:*** High knee march; sumo squat arms MB overhead; arm swing and lunge; backward hip flexion walk; superman; knee grab to lunge and twist; carioca; rocket jump; squat jump; lateral bound with stick; single-leg push-off; 90° sprint jump; back squat; bench press; medicine ball static lunge (bent arms); military press; horizontal pull-up; front squat; incline press; medicine ball static lunge (straight arms); behind neck press  ***CG:*** Regular routine |
| Katsikari et al. [82] | Plyometric training | Separate session | ***EG:*** Two-feet ankle hops; front cone hops; single-foot ankle hops; diagonal cone hops; single-foot side-to-side ankle hops; alternating push-off; side-to-side ankle hops two feet; single-leg push-off; side-to-side ankle hops one leg; lateral step-up; hip-twist ankle hops; side-to-side box shuffle; standing long jump (free hand); lateral shuffle to box; standing long jump (hands in the body); drop jump from box; standing jump and reach; jumps to box; standing jumps over barrier; step-close jump-and-reach; squat jump (free hands); skipping; squat jump (hands akibo); power skipping two hands; countermovement jump; power skipping alternating hand; hexagon drill (two feet); side skipping with big arm swing; hexagon drill (one foot); rim jump; block; front box jumps; jumps over barriers; lateral box jumps; box jumps; drop jumps (20 cm); drop from box and jump to next box; drop jumps from squat position (20 cm); drop jumps and catching a ball (20–30 cm)  ***CG:*** Regular content of the physical education lesson at school, including teaching of team-sport technical skills |
| Lim et al. [83] | Injury prevention training programme  (warm-up + flexibility + strength + plyometrics + agility training) | Prior to basketball practice session | ***EG:*** Jog line to line; shuttle run (side to side); backward running; calf stretch; quadriceps stretch; hamstring stretch; inner thigh stretch; hip flexor stretch; walking lunges; Russian hamstring; single toe raises; lateral hops over cone; forward/backward hops over cone; single-leg hops over cone; vertical jumps with headers; scissors jump; shuttle run with forward/backward running; diagonal runs; bounding runs  ***CG:*** Normal training routine |
| Otsuki et al. [84] | Injury prevention training programme  (strength + plyometric + COD training) | Warm-up | ***EG:*** Two-legged squat; one-legged squat; squat jumps; tuck jumps; 180˚ jumps; contact jumps; lateral jumps; pivoting; two-legged planting and cutting; one-legged planting and cutting  ***CG:*** Normal training routine |
| Otsuki et al. [85] | Injury prevention training programme  (strength + plyometric + COD training) | Warm-up | ***EG:*** Two-legged squat; one-legged squat; squat jumps; tuck jumps; 180˚ jumps; contact jumps; lateral jumps; pivoting; two-legged planting and cutting  ***CG:*** Normal training routine |
| Rojano Ortega et al. [86] | Plyometric training | Separate session | ***EG:*** Ankle hops; butt kick jumps; tuck jumps; hurdle jumps (25 cm); squat jumps; box jumps (25 cm); depth jumps (25 cm)  ***CG:*** Regular volleyball training sessions (warm-up and stretching routine, general strength training without weights, speed and agility drills and specific volley training) |
| Schmidt et al. [87] | Injury prevention training programme (balance training + plyometrics + jump and landing exercises + strength training) | Separate session | **EG:** Standing on stable surface (hall floor); standing on slightly unstable surface (e.g., rubber ring); standing on unstable surface (e.g., soft floor mat); standing on unstable surface with eyes closed or being destabilized by a partner; unidirectional double-leg jumps; multidirectional double-leg jumps; uni- and multidirectional single-leg jumps; uni- and multidirectional double- and single-leg jumps on a slightly unstable surface (e.g., stair of mats); isolated double-leg drop landings (25–40 cm height); isolated single-leg drop landings (25–40 cm height); isolated double- and single-leg drop with a rebound jump and second landing; series of double- and single-leg drop and rebound jump landings in horizontal direction over an obstacle (e.g., box); “standard” core exercises; core exercises with dynamic disturbances like lifting one leg or arm; core exercises with dynamic disturbances and one part of the body placed on an unstable surface (e.g., soft floor mat)  ***CG:*** Regular handball specific training |
| Sudds et al. [88] | FIFA 11+ injury prevention programme for dancers | First 30 mins of practice session | ***EG:*** Plank on elbow—raise leg; plank on hands—raise leg; plank on elbow—raise leg (opposite); plank on hands—raise leg (opposite); side plank—on knee; side plank—on elbow and side of foot; side plank—on hand and side of foot; single-leg bride and hold; hamstring raises; single-leg hamstring raises; squats to releve; squats to releve, slow eccentric phase; squats to releve, pause at bottom; walking lunges; demi-pointe to lunge; demi-pointe retire front to lunge; side lunges; side to cross-body lunges; single-leg balance—hip rotation; single-leg balance—hip rotation with closed eyes; single-leg balance—upper body rotation; single-leg balance—upper body rotation with closed eyes; cross-country skiing; cross-country skiing with overhead extension; hip airplane; hip airplane—increase height at leg; hip airplane—increase speed of movement; forward landings; forward landings—stick landings; forward landings—1 forward, 1 up; single-leg lateral landings; single-leg lateral landings—stick landing; single-leg lateral landings—1 forward, 1 up; plank on hands with elbow taps; plank on hands with shoulder taps; plank on hands and raise opposites; bear walk—static three-point contact; bear walk—forward/backward; bear walk—forward/backward/side; crab walk—static three-point contact; crab walk—forward/backward; crab walk—forward/backward/side; cook hip lift; cook hip lift—pause at the top; four-way monster walks—straight legs; four-way monster walks—demi-pointe; four-way monster walks—bend legs; squats with overhead reach; squats with overhead reach—hold dowel; squats with overhead reach—squeeze hands against each other; side slides; side slides—pause at bottom; leg swings forward; leg swings forward—increase speed; leg swings forward—closed eyes; leg swings cross-body; leg swings cross-body—increase speed; leg swings cross-body—closed eyes; single-leg reach; single-leg reach—go across body  ***CG***: Regular dance class schedule of three 90-minute ballet classes and two 60-minute modern classes per week; warm-up was self-directed consisting of static stretching and mobilization exercises |
| Taghizadeh Kerman et al. [5] | FIFA 11+ injury prevention programme | Warm-up | ***EG:*** Running, skating jumps, single-leg stance, press-ups, single-leg jumps, spiderman, and sideways roll  ***CG:*** Regular warm-up that included jogging, active stretching, and ball exercises such as dribbling and pass techniques |
| Thompson et al. [65] | F-MARC/FIFA 11+ injury prevention warm-up programme | Replace warm-up prior to practice | **EG:** six running exercises at moderate speed combined with dynamic stretching and controlled contact with a partner; six exercises targeting strength, balance, and jump-landing techniques with three levels of increasing difficulty; three high-speed running and cutting drills  ***CG:*** NR |
| Thompson-Kolesar et al. [66] | F-MARC/FIFA 11+ injury prevention warm-up programme | Replace warm-up prior to practice | ***EG:*** Front plank; side plank; Nordic hamstring; single-legged stance; squats; jumping  ***CG:*** NR |

***Abbreviations:*** CG­– control group; COD– change of direction; EG– experimental group; NR– not reported
